# Low pH structure of heliorhodopsin reveals chloride binding site and intramolecular signaling pathway

**DOI:** 10.1038/s41598-022-17716-9

**Published:** 2022-08-17

**Authors:** Jessica E. Besaw, Jörg Reichenwallner, Paolo De Guzman, Andrejs Tucs, Anling Kuo, Takefumi Morizumi, Koji Tsuda, Adnan Sljoka, R. J. Dwayne Miller, Oliver P. Ernst

**Affiliations:** 1 Department of Chemistry, University of Toronto, Toronto, ON M5S 3H6 Canada; 2 Department of Biochemistry, University of Toronto, Toronto, ON M5S 1A8 Canada; 3 Department of Laboratory Medicine and Pathology, University of Toronto, Toronto, ON M5S 1A8 Canada; 4 Graduate School of Frontier Sciences, The University of Tokyo, Kashiwa, Chiba 277-8561 Japan; 5 RIKEN Center for Advanced Intelligence Project, RIKEN, 1-4-1 Nihombashi, Chuo-ku, Tokyo, 103-0027 Japan; 6 Research and Services Division of Materials Data and Integrated System, National Institute for Materials Science, Tsukuba, Ibaraki 305-0047 Japan; 7 Department of Chemistry, York University, Toronto, ON M3J 1P3 Canada; 8 Department of Physics, University of Toronto, Toronto, ON M5S 3H6 Canada; 9 Department of Molecular Genetics, University of Toronto, Toronto, ON M5S 1A8 Canada

**Keywords:** Biochemistry, Biophysics, Computational biology and bioinformatics, Structural biology

## Abstract

Within the microbial rhodopsin family, heliorhodopsins (HeRs) form a phylogenetically distinct group of light-harvesting retinal proteins with largely unknown functions. We have determined the 1.97 Å resolution X-ray crystal structure of *Thermoplasmatales* archaeon SG8-52-1 heliorhodopsin (TaHeR) in the presence of NaCl under acidic conditions (pH 4.5), which complements the known 2.4 Å TaHeR structure acquired at pH 8.0. The low pH structure revealed that the hydrophilic Schiff base cavity (SBC) accommodates a chloride anion to stabilize the protonated retinal Schiff base when its primary counterion (Glu-108) is neutralized. Comparison of the two structures at different pH revealed conformational changes connecting the SBC and the extracellular loop linking helices A–B. We corroborated this intramolecular signaling transduction pathway with computational studies, which revealed allosteric network changes propagating from the perturbed SBC to the intracellular and extracellular space, suggesting TaHeR may function as a sensory rhodopsin. This intramolecular signaling mechanism may be conserved among HeRs, as similar changes were observed for HeR 48C12 between its pH 8.8 and pH 4.3 structures. We additionally performed DEER experiments, which suggests that TaHeR forms possible dimer-of-dimer associations which may be integral to its putative functionality as a light sensor in binding a transducer protein.

Heliorhodopsins (HeRs) are a newly discovered category of retinal-binding microbial rhodopsins^1,2^. Its first member, HeR 48C12, was discovered through functional metagenomics when the marine fosmid KIN48C12 yielded red *E. coli* colonies on retinal-containing culture plates^1^. This fosmid had low sequence similarity to known rhodopsins, which attributed to the lack of detection of HeRs in typical bioinformatic searches^1^. Now, 22 more HeRs have been discovered through pigmented *E. coli* screens^3,4^, and over 400 unique HeR sequences have been found from homology searches of whole genomes from microorganisms^5,6^. It turns out that HeRs comprise a large and diverse group of rhodopsins. HeRs are found in bacteria, archaea, eukaryota, and algal viruses^1,5,7,8^ (Fig. 1). They are globally distributed in marine, hypersaline, freshwater, and soil environments^1,7^. Moreover, HeRs can be found in psychrophiles, mesophiles, and hyperthermophiles that live in various temperature environments^1,8^. Interestingly, bioinformatic analysis revealed that HeRs are found in gram positive bacteria and are largely absent in gram negative bacteria^6,8^, although this taxonomic distribution in monoderms and diderms was somewhat debated^5^.

**Figure 1:**
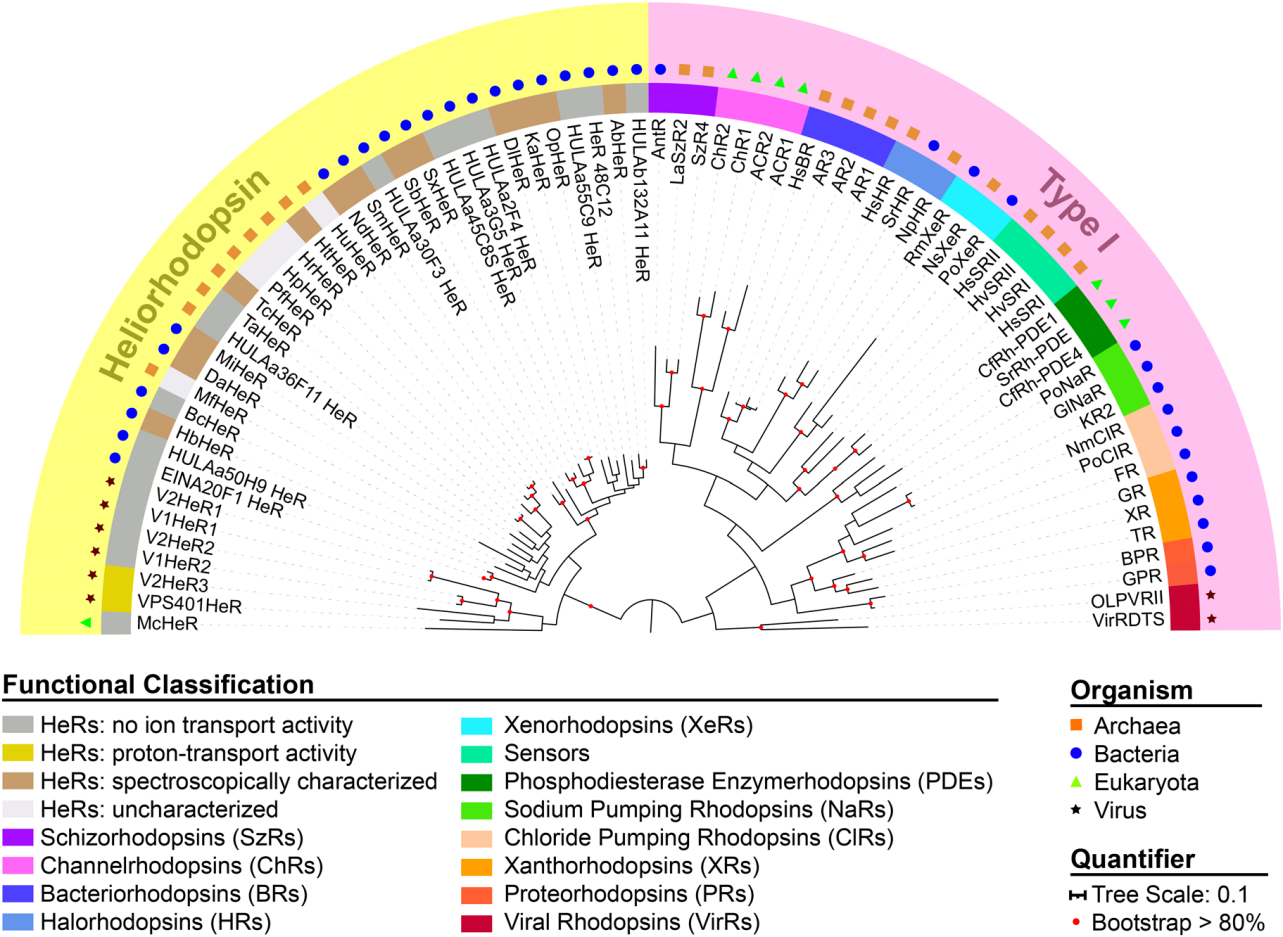
Phylogenetic tree of microbial rhodopsins with representative HeRs and type I rhodopsins. HeRs and type I rhodopsins form distinct branches arising from < 15% sequence identity. Microbial rhodopsins are widely distributed among archaea (orange squares), bacteria (blue circles), eukaryota (green triangles), and viruses (black stars). Type I rhodopsins have numerous diverse functions including outward H^+^ pumps (BRs, XRs, PRs, VirRs), inward H^+^ pumps (XeRs, SzRs), outward Cl^–^ pumps (HRs, ClRs), outward Na^+^ pumps (NaRs), anion and cation channels (ChRs), sensors, and enzymerhodopsins (including PDEs). A small group of viral HeRs show proton-transport activity (gold). Many HeRs have no ion transport activity (grey). Some HeRs that have been spectroscopically characterized only (brown) or are uncharacterized (white) are also shown. The tree scale bar shows the average number of amino acid substitutions per site. Red colored circles indicate bootstrap values > 80% for 100 replicates. See Supplementary Table S1 for the full names of proteins and sequence (NCBI searchable codes).

Despite their global abundance and growing scientific interest, the functions of HeRs remain elusive, although light-gated proton channel activity has recently been reported for a few HeRs^9^. This motivated further research to uncover the similarities and differences between HeRs and other rhodopsins. Rhodopsins are light-harvesting membrane proteins that are classified as either microbial rhodopsins (type I) or animal rhodopsins (type II)^10^. HeRs fall within the microbial rhodopsins category, but they are phylogenetically distinct from previously discovered type I rhodopsins (Fig. 1).

To date, several HeRs have been studied including HeR 48C12^1,5,7,11–15^, *Thermoplasmatales archaeon* SG8-S2-1 heliorhodopsin (TaHeR)^12,13,16^, *Bellilinea caldifistulae* heliorhodopsin (BcHeR)^3^, and more than 30 others that have been spectroscopically characterized or functionally investigated^1,4,9,16,17^. From experimental studies of these HeRs, it has become apparent that they share numerous structural and photochemical properties with other microbial rhodopsins. Similar to type I rhodopsins, HeRs possess seven-transmembrane α-helices (helix A to G) and covalently bind the all-*trans*-retinal chromophore at a conserved lysine on helix G through a Schiff base linkage^5,11,12,16^. In the ground state, the retinal Schiff base (RSB) is protonated (RSBH^+^).

HeRs and type I rhodopsins also have similar photoreaction dynamics^13^. In HeRs, the retinal chromophore isomerizes from all-*trans* to 13-*cis* with comparable ultrafast time scales (τ_isom_ = 0.42 ps for HeR 48C12 and τ_isom_ = 0.22 ps for TaHeR) and slightly lower isomerization quantum yields as type I rhodopsins^13^, which triggers a photocycle in the millisecond to second time frame. Both type I rhodopsins and HeRs pass through similar photocycle intermediates including K, M, and O^1,3–5,9^, although the M-intermediate was not observed for BcHeR^3^. The M-intermediate, a state in which the RSB is deprotonated, is also not observed in chloride-pumping microbial rhodopsins^10^. Thus, the lack of an M-intermediate for BcHeR may speak towards the functional diversity of HeRs. Further, as known for microbial rhodopsins, retinal adaptation depends on the protein. HeRs can either exhibit retinal adaptation like HeR 48C12 (dark-adaption: 97% all-*trans-*retinal; light-adaption: 40% all-*trans-*retinal)^1^, or no retinal adaptation like BcHeR (88% all-*trans*-retinal in both light and dark adapted states)^3^.

Despite these similarities, HeRs have unique sequences and topologies. First of all, HeRs share less than 15% sequence identity with other type I and type II rhodopsins^1,5,7,8^. More notably, HeRs have an inverted membrane topology compared to known microbial and animal rhodopsins^1,16^ (Fig. 2). HeRs expose their N-terminal tail towards the cytoplasm, while the N-terminus of type I and type II rhodopsins orient towards the outside of the cell.

**Figure 2:**
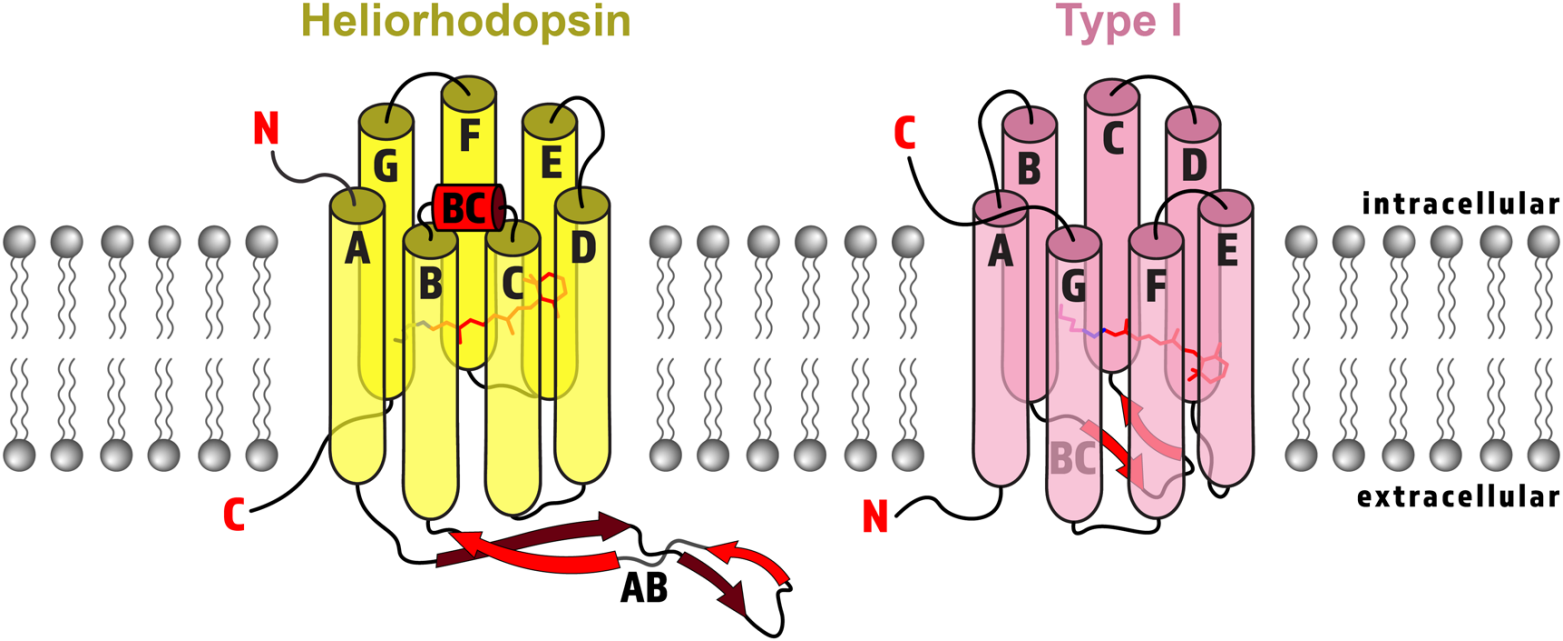
Cartoon structure representation of HeRs (yellow) and type I rhodopsins (pink) with major structural differences emphasized in red. Both HeRs and type I rhodopsins have seven transmembrane α-helices labelled A–G (except enzymerhodopsin, which has eight helices), with a covalently linked retinal chromophore (red sticks) bound to a lysine (pink or yellow sticks) by a Schiff base linkage (blue sticks) on helix G. HeRs have an inverted orientation in the membrane with the N-terminus (red N) facing inward and C-terminus (red C) facing outward, which consequently flips the retinal orientation. HeRs possess a long extracellular A–B loop (AB) composed of a twisted β-sheet (red arrows), and an intracellular B–C loop forming an α-helix (red cylinder labelled BC). In contrast, type I rhodopsins typically have an extracellular B–C loop forming a β-sheet.

There may be as many as 10 different sub-families of HeRs with potentially different functions^5^. A natural hypothesis is that HeRs may function like other microbial rhodopsins—as light-activated ion pumps, ion channels, light sensors, or enzymes. Very recently, three proton-transporting HeRs were discovered in marine giant viruses, including *Emiliania huxleyi* virus 202 (V2HeR3) and *Emiliania huxleyi* virus PS401 (VPS401HeR)^9^ as shown in Fig. 1. However, most known HeRs do not function as ion pumps or channels because they do not show light-dependent pH changes, including HeR 48C12^1^, BcHeR^3^, TaHeR^16^, ten HeRs discovered through functional metagenomics (EINA and HULA variants shown in Fig. 1)^4^, and other HeRs^1,9,16^. Interestingly, the photocycles for these HeRs (lacking ion-transport function) often exhibit a Schiff base deprotonation step (M-intermediate), but the proton does not exit the protein, not even transiently^1^.

The current theory is that many HeRs function as light-driven sensors. There is a plethora of evidence to support sensory function. First, long photocycles of greater than 1 s are observed for HeR 48C12^1^, TaHeR^16^, BcHeR^3^ and others^4^, which are characteristic for sensors like sensory rhodopsin I (SRI) and sensory rhodopsin II (SRII)^18–20^. In particular, the O-intermediate is long-lived in HeRs and a good candidate for a signalling state^1,3,4^. Secondly, HeRs are largely constrained to the photic zone—the surface layer of aquatic habitats that still receives enough sunlight for photosynthesis—which suggests that HeRs serve a light-harvesting function^1,4^. However, one exception is BcHeR which was discovered in thermophilic digester sludge which is an inherently low irradiance environment^3^. Thirdly, a resonance Raman study revealed that the vibrational mode of the RSB is like that of photosensory microbial rhodopsins^12^. Specifically, the RSBH^+^ of both TaHeR and HeR 48C12 forms a strong hydrogen bond to a glutamate residue^12^ with comparable strength to SRII from *Halobacterium salinarum*^21^ and *Natronobacterium pharaonis*^22,23^. If HeRs are indeed sensors, the transducer proteins have not yet been identified, because HeRs lack a consistent set of gene neighbours^4^. A wide genomic and metagenomic search provided compelling evidence that some HeRs are linked to histidine kinases, while other HeRs may be N-terminally fused to a MORN-repeat (Membrane Occupation and Recognition Nexus) or a Zinc-finger functional domain^6^. Another analysis of HeR-coding DNA segments revealed two protein families that were unique gene neighbors among some HeRs: Blh (β-carotene 15,15′-dioxygenase), required for the last step in the retinal biosynthetic pathway in prokaryotes; and the DegV family involved in activating fatty acids^4^. In order to resolve the biological role of HeRs, further functional and structural studies will be necessary.

The crystal structures of HeR 48C12^5,11^ and TaHeR^16^ have provided insight into the unique architecture and potential functions of HeRs (Fig. 2). The structures revealed that HeRs assemble as dimers with the most extensive dimerization interface seen in microbial rhodopsins (2260 Å^2^ for HeR 48C12)^11^. The most distinguishing feature of HeRs is the very long, extracellular loop linking helices A and B (~ 30 residues, ~ 40 Å in length) that forms two antiparallel β-strands, which is unique to HeRs^5,11,16^. This A − B loop is essential for dimer formation and HeR function, as its deletion causes protein aggregation and a complete loss of retinal binding^11^. On the intracellular side, an unusual B–C loop (~ 14 residues, ~ 18 Å in length) forms a structured α-helix^5^. Another striking element observed in both HeRs is a fenestration (an opening between helices towards the membrane) above the β-ionone ring of retinal, which has also not been observed in other rhodopsin structures^5,11,16^. For TaHeR, it was postulated that since its host organism lacks a means of producing retinal, the fenestration could facilitate binding exogenous retinal from the environment^16^.

Kovalev et al.^5^ crystallized HeR 48C12 in two different states: (1) a violet, basic form at pH 8.8 and (2) a blue, acidic form at pH 4.3, postulating that the low pH structure could give insight into conformational changes due to the protonation of the RSBH^+^ proton acceptor. The starkest difference between the pH 4.3 and pH 8.8 structures was observed in the large hydrophilic Schiff base cavity (SBC). Whereas the SBC was filled with seven water molecules at pH 8.8, it contained three water molecules and one acetate anion at pH 4.3^5^. Furthermore, key residues underwent pH-induced rearrangement which may indicate functionally relevant intramolecular signalling. In order to determine whether the observed structural changes between low and high pH forms are conserved among HeRs, we investigated the pH-induced structural changes of TaHeR. Using bicelle crystallization we determined the high resolution structure of TaHeR at pH 4.5, which revealed a chloride ion in the SBC and differences to the previously published pH 8 TaHeR structure^16^, arguing for a conserved intramolecular signalling behavior similar to that in HeR 48C12. This suggests that different sub-families of HeRs may potentially function as light sensors using a common mechanism.

Furthermore, we investigated the pH- and light-induced conformational changes of TaHeR in detergent using electron paramagnetic resonance (EPR) spectroscopy. EPR can provide course-grained site-specific structural information about the population of dynamic states of a spin-labelled protein in solution^24–26^. We explored the conformational dynamics of the spin-labelled A–B loop using continuous wave-EPR (CW-EPR) and double electron–electron resonance (DEER). CW-EPR detects the stabilized unpaired electron from a covalently attached spin label at a specific topological region of the protein and therefore provides local information on whether the spin label is mobile or restricted in motion^25^. DEER is an EPR technique in pulsed dipolar spectroscopy (PDS) that can be applied on a sample containing two or more spin labels. This method facilitates direct measurements of dipolar interactions in the range from typically 1.6–6.0 nm, or even up to 15.0 nm in optimized samples^27,28^. The resulting distance distributions yield information about structural changes, flexibility, and intermolecular alignments^27,29^. Thus, DEER constitutes a valuable alternative method to study rhodopsin oligomerization in solution^30^. Here, we utilize this integrative structural biological tool to access the intermolecular functional context of TaHeR that reaches beyond our high resolution data from X-ray crystallography^31^. Conveniently, since TaHeR is dimeric, only a single cysteine mutation is required for spin-labelling, and thus distances between spin labels on adjacent protomers can be obtained. This DEER study revealed that TaHeR can form transient dimer-of-dimer associations, akin to those observed in high-speed atomic force microscopy (HS-AFM) experiments^16^. We determined that these potential dimer-of-dimer associations are pH- and light-dependent, suggesting that the transient formation of dimer-of-dimers could be relevant to its functionality, say, as a sensory rhodopsin binding a transducer protein.

## Results and discussion

### Structure of TaHeR crystallized at low pH

**Figure 3:**
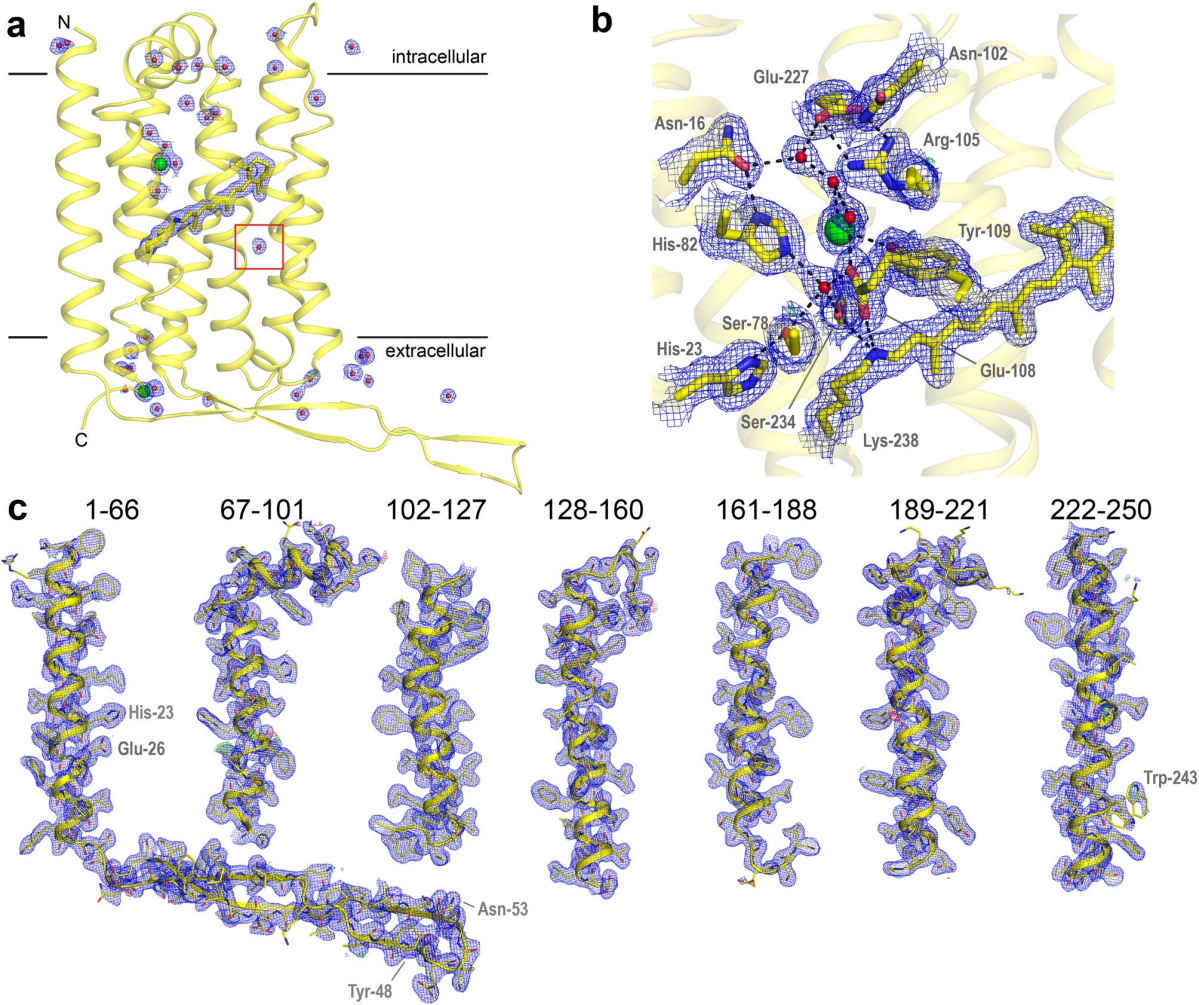
Electron density map and model of TaHeR crystallized at pH 4.5. (**a**) Asymmetric unit of TaHeR with electron density of water molecules (red spheres), chloride ions (green spheres) and retinal (yellow sticks). A red box highlights a single water molecule present in the highly hydrophobic extracellular half of TaHeR. (**b**) Electron density map of the SBC, with black dashed lines revealing a water-mediated hydrogen bonding network. (**c**) Electron density map of the protein backbone with the corresponding residue numbers listed above. Conserved residues in the suggested intramolecular signal transduction pathway have been labelled. For all images, the 2Fo − Fc map is contoured at 1 σ (blue mesh), while Fo–Fc is contoured at − 3 σ (red mesh) and in green at + 3 σ (green mesh).

Purified TaHeR (Supplementary Fig. S1) was crystallized using the bicelle method^32^ to yield large diamond shaped crystals (Supplementary Fig. S2). Notably, this is the first example of a HeR being crystallized via bicelles, whereas previously lipidic cubic phase crystallization^5,11,16,33^ was employed. We determined the TaHeR structure at 1.97 Å resolution (Table 1) using a single crystal grown at pH 4.5, enabling comparison with the other available TaHeR structure (PDB ID 6is6), which has been determined at 2.4 Å resolution using more than 100 crystals grown at pH 8 in lipidic cubic phase. The pH 4.5 TaHeR structure contains a single protomer in the asymmetric unit (Fig. 3a). It also possesses 37 water molecules, an all-*trans*-retinal chromophore covalently linked to Lys-238 (Fig. 3b), and two chloride ions. The electron density is well-resolved for all seven transmembrane helices and loops (Fig. 3c).

**Table 1:** TaHeR data collection and processing.

Sample name (PDB: ID)	TaHeR (PDB: 7u55)
**Data collection**	
Number of crystals	1
Diffraction source	APS beamline 23-ID-B
Wavelength (Å)	1.0331
Temperature (K)	100
Detector	Dectris Eiger 16 M
Crystal-detector distance (mm)	300
Rotation range per image (°)	0.2
Total rotation range (°)	90
Exposure time per image (s)	0.2
Space group	P2_1_2_1_2
*a*, *b*, *c* (Å)	89.85, 47.97, 56.69
α, β, γ (°)	90, 90, 90
Resolution range (Å)	47.94–1.97 (2.02–1.97)
Total no. of reflections	55,049 (2440)
No. of unique reflections	16,754 (1100)
Completeness (%)	94.4 (89.2)
Redundancy	3.3 (2.2)
〈*I*/σ(*I*)〉	4.3 (1.3)
*CC*_1/2_	0.98 (0.72)
R_merge_	0.13 (0.52)
Overall *B* factor from Wilson plot (Å^2^)	22.85
**Refinement statistics**	
Resolution range (Å)	47.94–1.97 (2.04–1.97)
Completeness (%)	93.3 (90.1)
No. of reflections, working set	16,733 (1583)
No. of reflections, test set	1671 (158)
*R*_work_/*R*_free_	0.198/0.223
No. of non-H atoms	2096
Protein	2025
Ligand	34
Water	37
R.M.S. deviations	
Bonds (Å)	0.008
Angles (°)	1.05
Average *B* factors (Å^2^)	28.26
Macromolecules	28.23
Ligand	24.42
Water	33.85
Ramachandran plot	
Most favored (%)	98.77
Allowed (%)	1.23
Outlier (%)	0.00

CC_1/2_ > 0.3 and 〈I/σ(I)〉 > 1.0 was used to determine cut-off. Values for the outer shell are given in parentheses.

The structures of TaHeR at pH 4.5 and pH 8 are very similar, with an RMSD of 0.535 Å with 220 aligned C_α_ atoms calculated using PyMOL. Both structures possess an asymmetric unit containing only one protein chain, and pack as a symmetric dimer. The higher resolution of the pH 4.5 structure revealed a single, isolated water molecule present in the highly hydrophobic extracellular half of TaHeR (Fig. 3a), which was not resolved in the pH 8 structure. Importantly, this water molecule can be also observed in both high resolution HeR 48C12 structures at pH 4.3 and pH 8.8 (Fig. 5b). The location of this water, close to the β-ionone ring of retinal and adjacent to the fenestration, appears to be conserved and unique to HeRs.

Furthermore, by comparing the two TaHeR structures, three key structural differences were observed. First, TaHeR at pH 4.5 binds two chloride ions while TaHeR at pH 8 has none. Second, the tail end of the A–B loop undergoes a 6 Å shift in position, and the B–C loop shifts ~ 1 Å. Finally, several conserved internal residues change orientation or become disordered. These differences provide valuable insights about anion binding sites and a potential intramolecular signalling pathway which are explored in the subsequent sections.

### Anions in the SBC under acidic conditions

This structural study of HeRs at different pH values gives insight into the interplay between the SBC and the counterion of the RSBH^+^. The previously published structure of TaHeR at pH 8 showed no negatively charged solvent ions bound to the protein^16^. This result is consistent with several ATR-FTIR studies showing wildtype TaHeR exhibits no specific binding of monovalent cations or anion at physiological pH (although the specific binding of Zn^2+^ was detected)^16,34^. In contrast, our pH 4.5 TaHeR structure revealed the presence of two chloride ions. One chloride resides in the SBC (Fig. 3b) and the other resides on the extracellular side adjacent to helices F and G (Fig. 3a).

**Figure 4:**
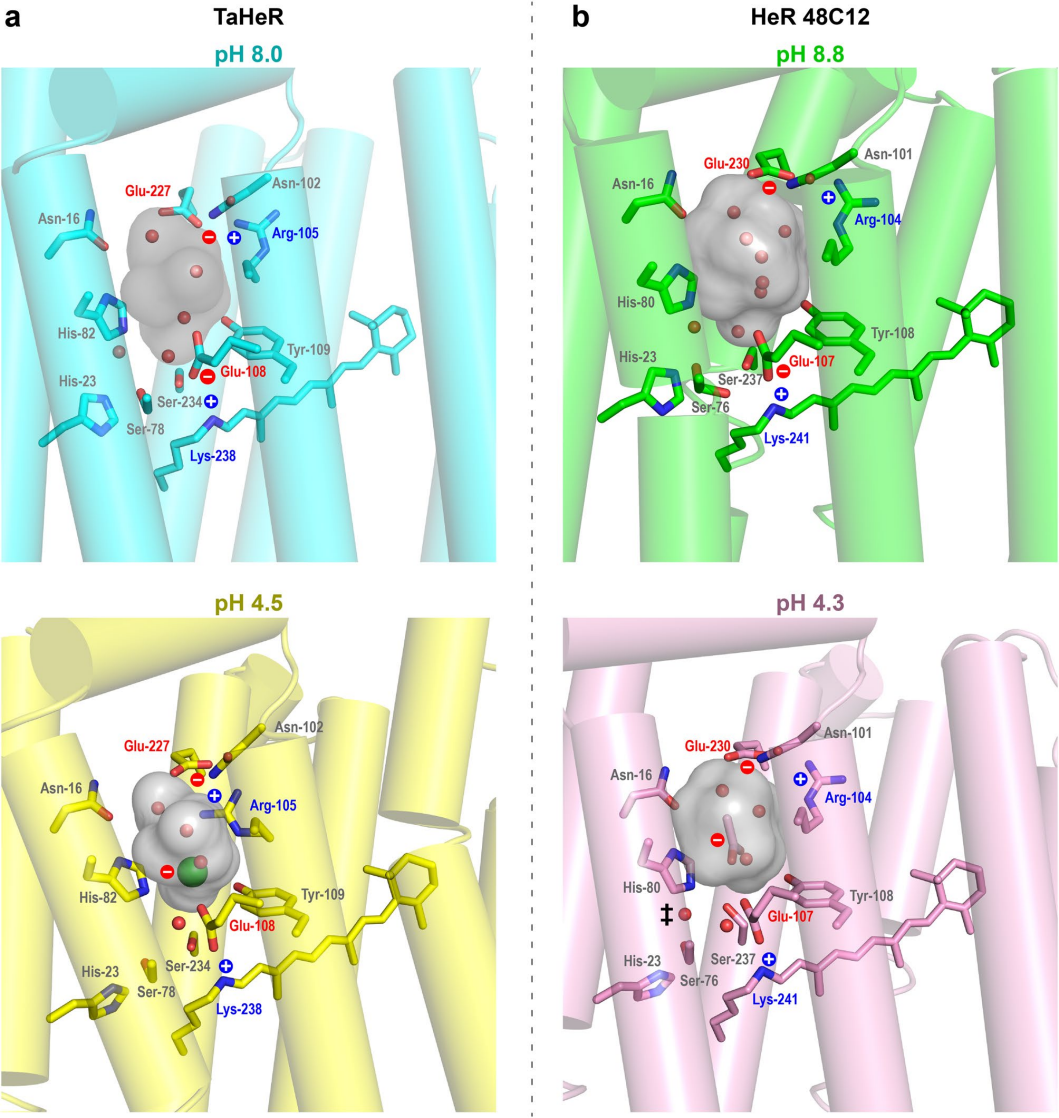
Schiff base cavity (SBC). SBC (grey surface) of (**a**) TaHeR and (**b**) HeR 48C12 under acidic and basic conditions. Under slightly basic conditions, the SBC of both TaHeR (PDB ID 6is6) and HeR 48C12 (PDB ID 6su3) contain only water molecules (red spheres) and are capped by two ionic interactions: (1) the RSBH^+^/Glu counterion pair and (2) the Arg/Glu pair. Under acidic conditions, the Glu counterion accepts a proton and is neutral. The SBC of TaHeR accommodates a negatively charged chloride ion (green sphere) while HeR 48C12 (PDB ID 6su4) accommodates a negatively charged acetate ion to maintain charge balance with the RSBH^+^. The negative charges are indicated by red dashes, while the positive charges are shown by blue pluses. ‡ Weak electron density adjacent to Ser-76 in the pH 4.3 HeR 48C12 structure has been modeled as a water molecule, but this molecule may also be absent, as in TaHeR.

Let us first explore the relevance of the chloride ion in the SBC. TaHeR possesses a large hydrophilic SBC on the intracellular half of the transmembrane region. The SBC is surrounded by numerous polar residues and bookended by two electrostatic interactions. As shown in Fig. 4a, at pH 8 the SBC of TaHeR accommodates four water molecules in a volume of 189 Å^3^ (calculated using Hollow^35^), and possesses two ionic interactions: (1) the RSBH^+^/Glu-108 pair, and (2) the Arg-105/Glu-227 pair. The surrounding histidine residues, His-23 and His-82, are assumed to be neutral because there are no other negatively charged residues in this space^16^. In the pH 4.5 TaHeR structure, the SBC shrinks to a volume of 156 Å^3^ and accommodates a chloride ion and three water molecules. The uptake of the chloride ion suggests that a positive charge has also been introduced, presumably by the RSBH^+^ counterion Glu-108, which becomes neutralized at the low pH. The protonation of Glu-108 is consistent with the observed 18 nm red-shift of TaHeR at low pH (Supplementary Fig. S3 and Shihoya et al.^16^). Acid-induced chloride ion binding is also present in solution, where TaHeR at pH 5.0 underwent a 12 nm red-shift, from 543 nm in a chloride-depleted form to 555 nm at 300 mM NaCl, with an estimated K_d_ of 67 mM (Supplementary Fig. S4). Coincidently, the counterion mutant, TaHeR-E108A with a neutral side chain, exhibits the same λ_max_ in the presence of 100 mM NaCl^16^. Our low pH structure suggests that a chloride anion in the SBC can serve as a stabilizing counterion to the RSBH^+^ when the Glu-108 counterion has been neutralized by protonation at low pH or by mutation.

A similar result was obtained from HeR 48C12 structures (PDB ID 6su3, 6su4), which possess the same polar residues surrounding the SBC as TaHeR. At pH 8.8, HeR 48C12 has a SBC of 227 Å^3^ (calculated with chain A of the structure) that accommodates seven water molecules (Fig. 4b). At pH 4.3, its SBC shrinks to 216 Å^3^ and accommodates a negatively charged acetate ion and three water molecules induced by the protonation of its Schiff base counterion, Glu-107^1^. Singh et al.^15^ showed that HeR 48C12 wildtype does not bind specific anions, whereas the counterion neutralizing mutants, E107A and E107Q, both bind anions at pH 7.0, which are needed to keep the RSBH^+^ protonated. The E107A mutant of HeR 48C12 in the presence of 500 mM chloride shows the same λ_max_ as its wildtype^15^, similar to TaHeR. The authors proposed that since E107A absorbance exhibited a large ion dependence (12 nm redshift from chloride to iodide), the anion may be directly hydrogen bonded to the RSBH^+^. In contrast, the E107Q mutation shows small ion dependence (2 nm redshift from chloride to iodide), suggesting that the anion may be hydrogen bonded to the N–H group of Glu-107. This hypothesis can now be specified by our pH 4.5 TaHeR structure, where the analogous protonated O–H group of Glu-108 is located 4.6 Å from the chloride anion and linked by two hydrogen bonded water molecules (Supplementary Fig. S3).

Given the same behavior for TaHeR and HeR 48C12 and the well conserved amino acids enveloping the SBC among the HeR family, we conclude that upon replacement of the primary counterion with a neutral residue or under acidic conditions, most HeRs will likely accommodate exogenous anions as surrogate counterions to stabilize RSBH^+^. This behavior has also been observed in type I rhodopsins, like the outward-proton pump bacteriorhodopsin (BR). Wildtype BR does not bind chloride. However, BR was found to bind anions from solution to stabilize the RSBH^+^ in the D85S and D85T single counterion mutants^36,37^, the D85N/D212N double counterion mutant^38^, and at very low pH in the acid-purple form^39^.

### A putative intramolecular signal transduction pathway

If HeRs function as sensors, they must have a mechanism to propagate conformational changes to a transducer protein. It was previously shown for TaHeR prepared with a “locked” all-*trans*-retinal (where isomerization is prevented by a 5-membered ring) that light-induced protein conformational changes occur even in the absence of retinal isomerization, which are instead triggered by the retinal excited state^40^. In a similar way, pH changes have also been shown to induce conformational changes due to protonation or deprotonation of key residues, which may reflect possible rearrangements in protein structure during its photocycle^5,41^. By comparing the available HeR structures at different pH values, we observe a conserved propagation of conformational changes originating from protonation of the RSBH^+^ counterion, which we later demonstrate computationally (see below).

**Figure 5:**
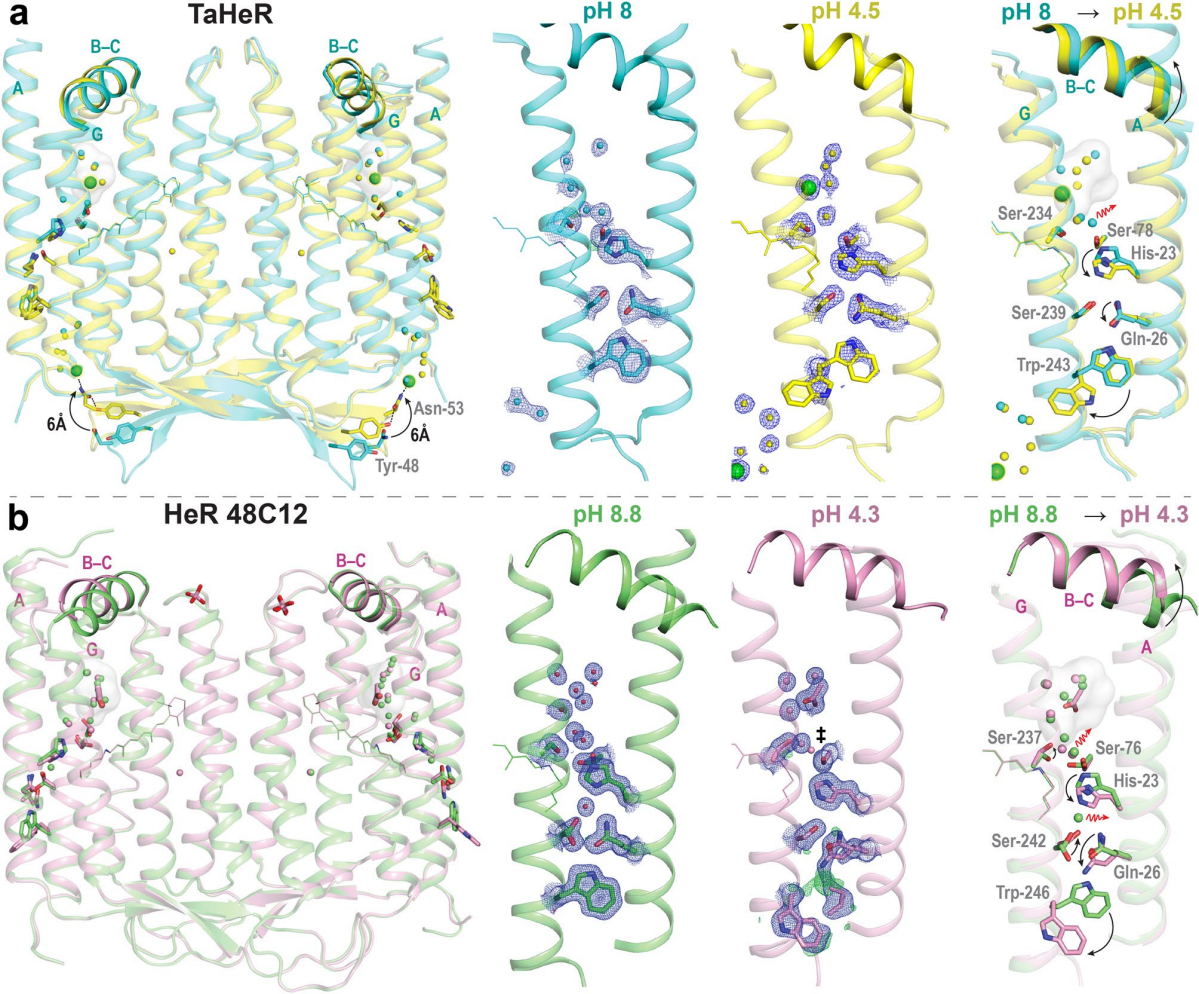
A putatively conserved intramolecular signaling pathway for TaHeR and HeR 48C12. (**a**) TaHeR dimer structure at pH 8 (cyan, PDB ID 6is6) and pH 4.5 (yellow, PDB ID 7u55). (**b**) HeR 48C12 dimer structure at pH 8.8 (green, PDB ID 6su3) and pH 4.3 (pink, PDB ID 6su4). The conserved intramolecular signaling mechanism involves a negatively charged anion (chloride or acetate) occupying the SBC (grey surface), switching the orientation of His-23, displacing water molecules (red arrows), disordering Gln-26 and disordering Trp-243 (in TaHeR) or Trp-246 (in HeR 48C12). The overlay reveals there is a 1 Å movement of the intracellular B–C loop. TaHeR has an additional 6 Å shift of the A − B tail from the electrostatic attraction of Tyr-48 and Asn-53 to an external chloride ion. Waters are shown as small spheres colored to match protein, and chloride ions as large green spheres (belonging to pH 4.5 TaHeR only). The 2Fo − Fc maps (blue mesh) are contoured at 1 σ, and Fo − Fc is contoured at + 3 σ for positive (green mesh) and − 3 σ for negative (red mesh) electron density, respectively. ‡ In HeR 48C12 at pH 4.3, there is no electron density to account for this water in the chain A protomer.

Previously, Kovalev et al. compared the pH 4.3 and pH 8.8 structures of HeR 48C12 and observed two main changes, which are depicted in Fig. 5b. Adjacent to the RSB, residues Ser-76 and Ser-237 underwent rearrangement. Further, in the basic form, there exists a hydrogen-bonded network on the extracellular half of the protein comprising a water molecule, Ser-242, Gln-26, and Trp-246 linking helices A and G. In the acidic form, the adjacent His-23 rotates and displaces the water to induce a large conformational change in the network, flipping the orientation of Trp-246 and breaking the Glu-26–Trp-246 hydrogen bond. It was proposed that if HeRs are light sensors, Trp-246 reorientation may trigger interaction with a signal transducer protein^5^.

By comparing the pH 8 and pH 4.5 TaHeR structures (Fig. 5a), we see very similar behavior to HeR 48C12. His-23 shows a well-defined reorientation, a water molecule adjacent to Ser-78 is displaced, and Trp-243 and Gln-26 become disordered by occupying two conformations rotated ~ 180° apart. A difference to HeR 48C12 is that the backbone of TaHeR at both pH are well aligned, except at the apex of the large A–B loop encompassing residues 46–56. At its largest, there is a ~ 6 Å shift between the C_α_ atoms of Ile-51. This A–B loop movement observed between the two TaHeR structures may originate from the different crystallization methodology or different crystal packing. In the low pH structure, the side chain nitrogen of Asn-53 is electrostatically attracted by the extracellular chloride ion in the other protomer and the sidechain oxygen of Asn-53 is hydrogen bonded to Tyr-48. These polar interactions pull the tip of the A–B loop 6 Å closer to the other protomer at residue Ile-51. Out of the four compared low/high pH structures of TaHeR and HeR 48C12, the pH 8 TaHeR structure is different. It has the lowest resolution and shows three-times larger B-factors for the tip of the A–B loop than for the overall structure. The observed A–B loop movement may be explained by a reduction in loop dynamics as a consequence of crystallization, which motivated us to apply computational methods and EPR/DEER spectroscopy.

The A–B loop movement is not observed in HeR 48C12, perhaps since its pH 4.3 and pH 8.8 structures were both crystallized using LCP and possessed similar crystal packing. Additionally, HeR 48C12 has different residues comprising the bend in the A–B loop. HeR 48C12 has a non-polar Gly-Pro-Pro-Gly (GPPG) motif, while TaHeR has a polar Tyr-Asp-Glu-Ile (YDEI) motif, both spanning residues 48–51 in the A–B loop. As discussed above, the first residue in the YDEI motif, Tyr-48 in TaHeR, may contribute to the A–B loop movement. The GPPG motif is conserved among many HeRs including those in *Ornithinimicrobium pekingense* (OpHeR), *Nocardioides dokdonensis* (NdHeR) and *Demequina lutea* (DlHeR)^17^. The polar motif is more variable with FDEA, FDTT, FDEI, and other variants in HeRs from *Thermococcus* sp. 2319 × 1 (TcHeR), *Halorhabdus tiamatea* SARL4B (HtHeR), and *Dehalogenimonas alkenigignens* (DaHeR)^17^, respectively. It was previously shown that mutating GPPG to FDEA in OpHeR yielded a + 1 nm redshift of the maximum retinal absorbance, while the reverse FDEA to GPPG mutation in TcHeR yielded a + 3 nm redshift^17^, suggesting that disrupting the polar motif causes conformational changes which reach to the retinal binding pocket/RSB to affect the HeR's absorption properties.

### Allostery and dynamics in HeRs

**Figure 6:**
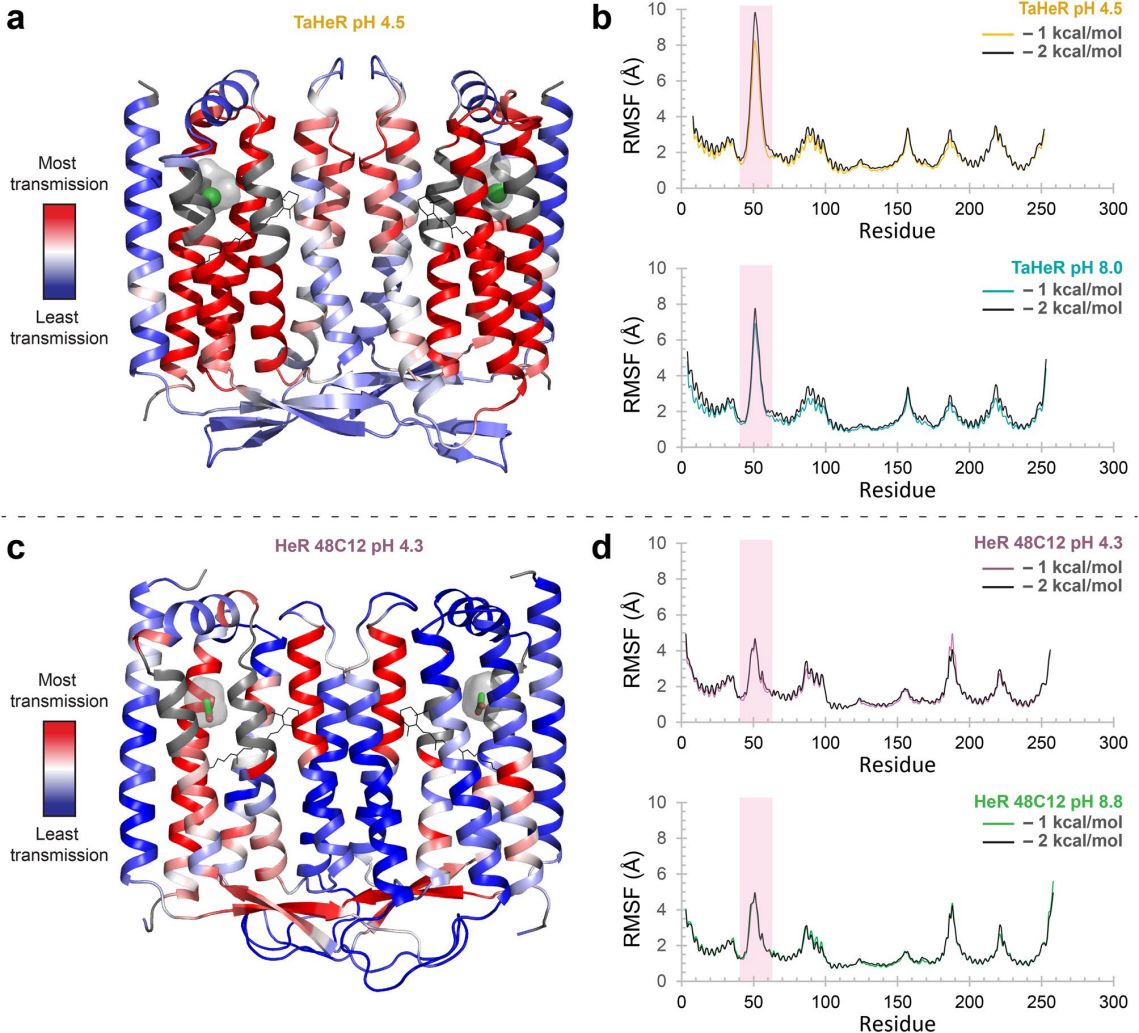
Allosteric transmission and conformational dynamics modelling in TaHeR and HeR 48C12 dimers. (**a**, **c**) The allosteric network within HeR dimers is revealed through rigidity theory allostery analysis. Allosteric transmission is measured by changes in conformational degrees of freedom (red/blue gradient bar) experienced upon rigidification of the negatively charged ion (chloride or acetate, green) occupying the SBC (grey surface) of the acidic pH structure. Amino acids within 3 Å of the charged ion (grey portions of the helix) were omitted from the coloring scheme to prevent skewed coloring biases, as neighbouring residues would naturally have high allosteric effects. (**b**, **d**) Constrained geometric Monte Carlo-based dynamics analysis predicts individual residue dynamics measured as the root mean squared fluctuation (RMSF). The extracellular β-sheet (A–B loop) that was further on investigated by DEER is highlighted in pink.

We investigated how a charge perturbation in the SBC base cavity of HeRs could facilitate signal transmission using rigidity transmission allostery (RTA) algorithms^42,43^. RTA utilizes concepts in mathematical rigidity theory^42–46^ to analyze allosteric networks within protein structures. RTA is based on the idea that a binding event at one site introduces constraints which perturb local conformational degrees of freedom and changes in rigidity that could propagate and cause a change in degrees of freedom in distant parts of a structure. The allosteric communication is characterized by significant changes in degrees of freedom. Upon rigidifying the anion in the SBC, chloride for TaHeR and acetate for HeR 48C12, an allosteric pathway across the membrane emerges (Fig. 6a,c). An allosteric pathway can be defined between the perturbed SBC and the A–B and B–C loops in the extracellular and intracellular space, respectively, traversing a central four-helix bundle comprising transmembrane helices B, C, F, and G for both HeRs. Interestingly, applying RTA to the SBC of one protomer leads to allosteric changes propagating to the other protomer, suggesting that allostery is enhanced by the dimer (data not shown). Ultimately, if HeRs behave as light sensors, the SBC may be critical in facilitating allosteric transmission for signaling.

Constrained geometric Monte Carlo simulations reveal that the A–B loop is the most dynamic portion of TaHeR and HeR 48C12 (Fig. 6b,d). TaHeR exhibits a greater root mean squared fluctuation (RMSF) in the A–B loop than HeR 48C12. Since the A–B loop in HeR 48C12 retains more rigidity and possesses more optimal interaction with the rest of the protein, allosteric transmission can propagate further into the β-sheet (Fig. 6c, red β-sheet) compared to TaHeR (Fig. 6a, light blue β-sheet). The large amplitude dynamics are somewhat unexpected since the A–B loop in HeR is essential in dimer formation. A higher amplitude of motion may be required to facilitate unlatching of the A–B loop for a light-activated function. In a previously published HS-AFM video of HeR 48C12^16^, two dimers can be observed interacting as a pseudo tetramer, where they exchange protomer partners and then dissociate back into dimers (Supplementary Fig. S5). This oligomeric exchange would require that the A–B loop possesses sufficiently high dynamics to switch binding partners by detaching from one protomer and binding to another.

### Electron paramagnetic resonance spectroscopy

The pH 4.5 and pH 8.0 TaHeR structures suggested that the A–B loop conformation may play a role in the TaHeR photocycle. Thus, the dynamics of the A–B loop subject to different pH and illumination conditions were explored using CW-EPR and DEER.

**Figure 7:**
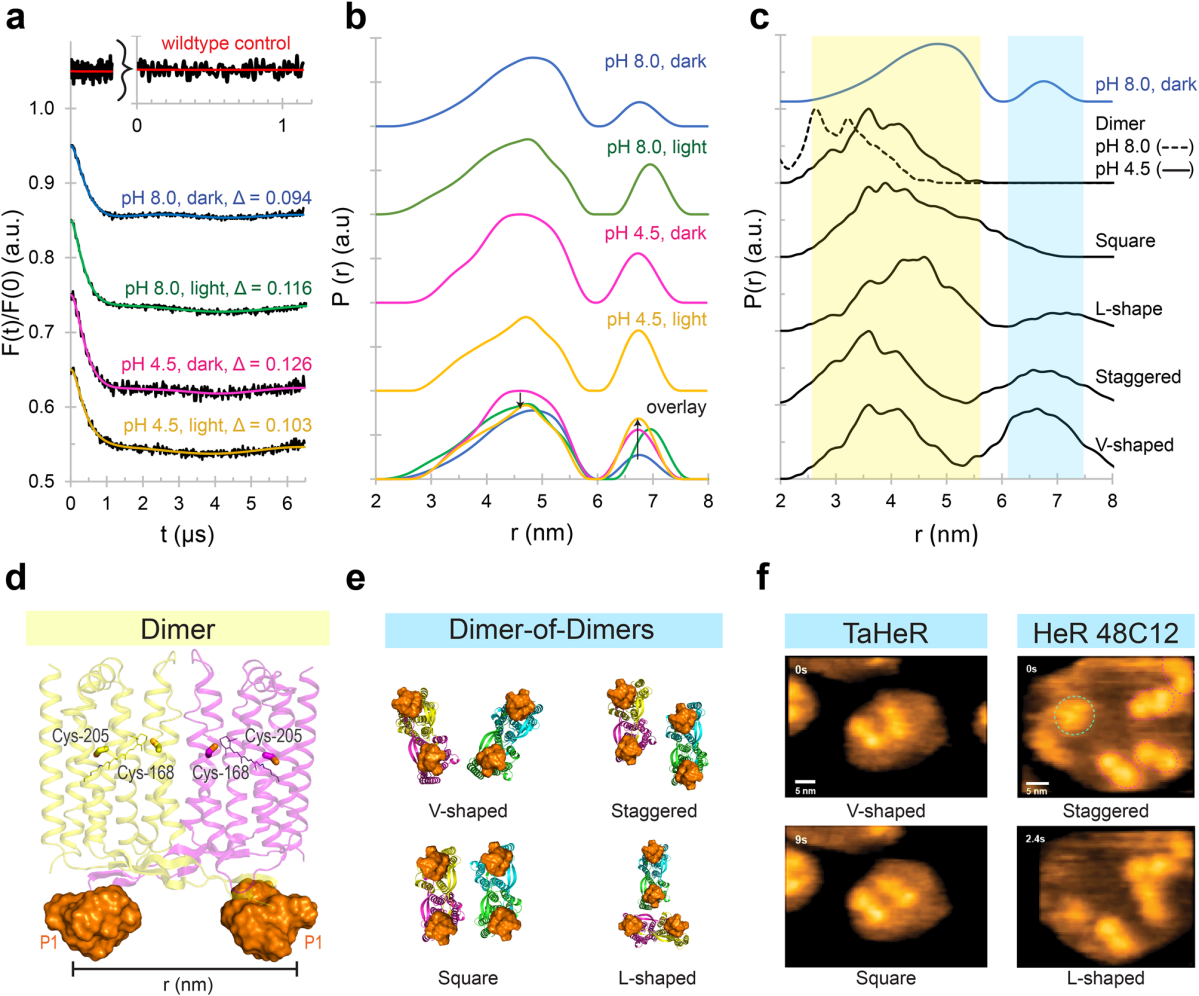
DEER of TaHeR-I51P1 suggests HeR can adopt dimer-of-dimer assemblies that are pH- and light-dependent. (**a**) The DEER dipolar evolution function of TaHeR-I51P1 with indicated modulation depth (Δ) at pH 8 and pH 4.5 in dark and light conditions. The inset (red) shows data from wildtype TaHeR incubated with IAP spin label. Here, the lack of dipolar interactions indicated that native cysteines could not be sufficiently spin-labelled. (**b**) The DEER distance distribution, P(r), revealed two spatially separated peaks at 4.7 nm and 6.6 nm. The experimental parameters allow for accurate distance distributions up to 5.9 nm and mean distances up to 7.4 nm^24^. Relative to the pH 8 dark sample, light or acidification increases the population of the 6.6 nm peak (up arrow). Relative to the pH 4.5 dark sample, illumination slightly decreases the population of the 4.7 nm peak (down arrow). DEER experiments were performed in duplicate. (**c**) MMM distance distributions of dimer and dimer-of-dimer assemblies compared to the pH 8 dark sample. Dimer-of-dimer assemblies were generated from AlphaFold (square, L-shaped) or PyMOL (staggered, V-shaped), with corresponding models shown in panel e. (**d**) Model of the TaHeR-I51P1 dimer with the P1 rotamer cloud (orange surface) from the pH 4.5 structure. The two native cysteines, Cys-168 and Cys-205, are inaccessible to spin-labelling. (**e**) Dimer-of-dimer models with P1 rotamer clouds (orange surface). (**f**) HS-AFM images of TaHeR and HeR 48C12 from the supplementary videos of ref.^16^ showing similar dimer-of-dimer assemblies. Reprinted by permission from CCC: Springer Nature Shihoya et al. (2019)^16^.

For EPR experiments, Ile-51 was mutated to cysteine (TaHeR-I51C) and then combined with a cysteine-reactive spin label, MTSL (1-oxyl-2,2,5,5-tetramethyl-∆3-pyrroline-3-methyl methanethiosulfonate) or IAP (3-(2-iodoacetamido) proxyl)^47^. Native cysteines, Cys-168 and Cys-205, were not accessible for the spin labels as shown by two control experiments (Fig. 7a and Supplementary Fig. S7). This can be accounted for by topological shielding of the detergent micelles. Since TaHeR-I51C initially formed unwanted covalent oligomers via Cys-51 linked disulfide bonds, we designed a purification protocol that successfully reduced covalent oligomers and selected for spin-labelled dimers (Supplementary Fig. S6).

CW-EPR revealed that TaHeR-I51C was successfully spin-labelled with MTSL or IAP (Supplementary Fig. S7), forming TaHeR-I51R1 or TaHeR-I51P1, respectively. Moreover, CW-EPR spectra of TaHeR-I51R1 and TaHeR-I51P1 show relatively narrow linewidths, which are characteristic for a spin label attached to a flexible loop region^25^, as expected. The spectral shape was largely unaffected by changes in pH or light exposure, suggesting no major changes in secondary structure, polarity, or solvent exposure around this label. Because the R1-labelled samples precipitated under acidic conditions, we focused on the P1-labelled samples, which gave consistent and reproducible data for all tested conditions.

Thereafter, DEER was employed to yield distance distributions between spin labels on adjacent A–B loops in the TaHeR dimer. The DEER experiment on TaHeR-I51P1 at pH 8.0 showed a rather broad distance population centered at ~ 4.7 nm and a peak at 6.6 nm that could be only clearly separated and identified after sample perdeuteration for better long-distance resolution (Fig. 7b). We assume that the broad feature in P(r) highlights highly flexible loop dynamics that are blurring out further conformational details. Based on the computed DEER distributions using MMM software^48^, employing our TaHeR structures at pH 8 and 4.5, we expected to observe distances only between 2 and 5 nm for both pH values, with a shift towards larger distances under acidic conditions (Fig. 7c). Initially, an unexpected long distance beyond this predicted distance range was also measured for TaHeR-I51R1 (Supplementary Fig. S8). The long-distance peak at 6.6 nm is already visible in the dipolar evolution function, appearing as a low frequency dipolar oscillation that persists even after background correction. The presence of this oscillation cannot be explained by the sample concentration of 220 µM, which would give rise to an estimated mean distance of ca. 25 nm in between individual TaHeR dimers (see Supplementary methods for calculation).

For TaHeR-I51P1, beyond the effect of pH (4.5, 8.0), also the effect of illumination (dark, light) on the A–B loop was investigated. The distance distributions for all samples exhibited similar shapes with slightly varying peak populations (Fig. 7b), which we further validated (Supplementary Fig. S9). The DEER data did not show a clear pH-induced shift to larger distances as predicted from MMM, which reflects the wide ensemble of conformations available to the flexible A–B loop in solution. This dynamic feature may be inaccessible in the crystal due to packing constraints and constitutes a major advantage of applying DEER spectroscopy in general^31^. MMM also did not account for the 6.6 nm population. For a standalone dimer (Fig. 7d) to achieve this long distance, the A–B loops would need to undergo extremely large conformational changes.

As an explanation for this long distance, we propose that TaHeR can form transient dimer-of-dimers associations. These pseudo-tetramers could account for the broad long-distance peak at 6.6 nm. This dimer-of-dimers hypothesis is further supported by slight changes in the modulation depth (Δ, Fig. 7a), which can serve as an indirect measure for the number of coupled spins per cluster^24,29,49,50^. Relative to the pH 8.0 dark sample (Δ = 0.094), there was an increase in the modulation depth under illumination (Δ = 0.116), acidification (Δ = 0.126), or both (Δ = 0.103), denoting an increase in the number of spins per cluster and therefore a slight change in oligomerization can be anticipated. According to our data, an increase in modulation depth is generally also accompanied by an increase in the 6.6 nm peak population. Thus, light irradiation, or lowering pH could slightly increase the number of dimer-of-dimer assemblies. Unusually, illumination of the acidic dark sample decreases its modulation depth, while still increasing the 6.6 nm distance peak. We can conclude that light exposure and protein net charge can be both used to induce slight variations in the intermolecular dimer-of-dimers assembly of TaHeR.

The idea of ‘dimer-of-dimers’ is also strongly supported by high-speed atomic force microscopy (HS-AFM) as performed on TaHeR and HeR 48C12 reconstituted into lipid bilayers^16^. HS-AFM of TaHeR at pH 8 shows prolonged dimer-of-dimers interactions oriented in a V-shape or square shape^16^ (Fig. 7f). Similarly, HS-AFM of HeR 48C12 showed transient dimer-of-dimers associations that existed in an L-shaped, staggered shaped, or V-shaped alignment^16^ (Fig. 7f). Both phenomena can be observed throughout the full HS-AFM recording, although not explicitly discussed in the publication.

Dimer-of-dimer models were generated from either the X-ray crystal packing, Alphafold^51,52^, or structural modelling using PyMOL^53^. MMM was used to simulate distance distributions from these pseudo tetramers, all of which are all presented in Supplementary Fig. S10, and the best fit assemblies in Fig. 7c. Briefly, the crystallography-derived pseudo tetramers, including vertically stacked and inverted-L shaped, did not fit the DEER distribution and are also considered as biologically irrelevant arrangements in the cell membrane. AlphaFold predicted more biologically plausible L-shaped and square-shaped tetrameric assemblies (Fig. 7e), with all protomers oriented in the same plane and membrane orientation. The L-shape conformation fits the DEER data exhibiting appropriate short and long distances, while the square-shaped alignment only populates the short distances (Fig. 7c). Finally, PyMOL was employed to generate staggered and V-shaped tetramer orientations of TaHeR (Fig. 7e) based on the conformations observed in the aforementioned HS-AFM videos^16^. Both V-shaped and staggered orientations fit the experimental DEER data, resembling the short and long-distance features quite well (Fig. 7c).

Ultimately, the combination of DEER, structural modelling, and MMM computations revealed that TaHeR-I51P1 exhibits long distance dipolar interactions from potential dimer-of-dimer assemblies, and short distance dipolar interactions from a convolution of dimer and pseudo tetramer contributions. These potential dimer-of-dimer assemblies occur even in detergent micelles and are pH- and light-dependent. Specifically, light and acidification promote dimer-of-dimer assemblies increasing the population of the long distance peak at 6.6 nm. We propose that these dimer-of-dimer assemblies may be necessary for a putative light sensory function, such that two TaHeR dimers adopt a specific relative orientation to bind a transducer protein.

In summary, the investigation of TaHeR using X-ray crystallography, computational methods, and DEER enabled us to identify an anion binding site that compensates for the neutralization of the Schiff base counterion at low pH, propose a conserved intramolecular signaling pathway, illustrate a mechanism for allosteric signal transmission, and identify higher order assemblies of heliorhodopsin in detergent. This work provides insights into conformational changes of heliorhodopsin that might be used by the microbial rhodopsin to serve as a possible light sensor.

## Methods and materials

### HeR expression and purification

For expression of TaHeR wildtype, a gene encoding *Thermoplasmatales* archaeon SG8-52–1 HeR (GenBank ID: KYK26602.1) with an N-terminal 6 × His tag was obtained from GenScript and cloned into the pET21a(+) vector using NdeI-XhoI restriction sites. For expression of mutant TaHeR-I51C, codon exchange from ATC to TGC was done using the QuikChange Lightning (Agilent) toolkit. The mutation was verified by DNA sequencing (ACGT Corporation, Toronto). TaHeR was expressed in *E. coli* OverExpress™ C43(DE3) chemically competent cells using a similar protocol described previously^16,33^. Transformed *E. coli* cells were grown (37 °C, 200 rpm) in LB media supplemented with 100 µg/mL ampicillin until an OD_600_ of 0.6 was obtained. Protein expression was induced by the addition of 0.5 mM isopropyl-β-D-thiogalactopyranose and 5 µM of all-*trans* retinal, and incubated overnight at a reduced temperature (25°, 200 rpm, 20 h). The collected cells were lysed using an Emulsiflex C3 (Avestin) in buffer containing 50 mM MES, pH 6.5, 300 mM NaCl, and a SIGMAFAST™ protease inhibitor cocktail tablet (Sigma). The crude membranes were pelleted via ultracentrifugation (4 °C, 1 h, 130 000× *g*, Beckman, 45Ti rotor), and then solubilized in 50 mM MES, pH 6.5, 300 mM NaCl, 2% w/v of n-octyl β-D-glucopyranoside (OG, Glycon) overnight at 4 °C. Solubilized TaHeR was recovered through ultracentrifugation (4 °C, 1 h, 130 000 × g) and the pellet was discarded. By approximating ε_550_ = 42,100 M^−1^ cm^−1^ at pH 6.5, the yield of TaHeR was ~ 10 mg per litre of expression medium.

A two-step purification protocol of nickel immobilized metal affinity chromatography (Ni^2+^ IMAC) and size exclusion chromatography was employed to achieve TaHeR at > 95% purity as seen by gel electrophoresis and western blotting (Supplementary Fig. S1). For IMAC purification, the solubilized solution was loaded onto a HisTrap™ 1 mL Ni–NTA column (GE Healthcare) equilibrated with 20 mM imidazole, 50 mM MES, pH 6.5, 300 mM NaCl, 1% OG. TaHeR was eluted using a 30-column volume linear imidazole gradient from 20 to 500 mM imidazole. Fractions with sufficient purity (A_550nm_/A_280nm_ > 0.65) were combined and concentrated using a 30 kDa concentrator tube (Amicon). Size exclusion chromatography (SEC) was run on 1 mL of concentrated protein loaded on a Superdex™ 200 10/300 GL column (Cytiva) using 20 mM MES, pH 6.5, 300 mM NaCl, 1% OG. Consecutive fractions with A_550nm_/A_280nm_ > 0.7 were combined and prepared for crystallization.

### Crystallization and harvesting

24% w/v bicelles composed of 2.8:1 DMPC:CHAPSO (molar ratio) were prepared in advance^32^, and stored at –20 °C. Prior to crystallization, the bicelles were thawed at room temperature and then kept on ice. Purified TaHeR was concentrated to 7.5 mg/mL and combined with bicelles in a 2:1 ratio (resulting in 5 mg/mL protein: 8% bicelle), then kept on ice for two hours to encourage mixing. The crystallization buffer was prepared containing 26% polyethylene glycol 3350, 0.1 M sodium phosphate monobasic monohydrate at pH 4.5 (Hampton Research), 0.28 M ammonium sulfate, and 0.18 M 1,6-hexanediol. Hanging-drop vapour-diffusion crystallization experiments were set up on standard pre-greased 24-well crystallization trays (Hampton). 4 µL of TaHeR-bicelle mixture was gently added to 1.5 µL of crystallization buffer without mixing on a thick siliconized cover slide, which was held over 0.5 mL of crystallization buffer in the reservoir. Crystal trays were stored at 34 °C and left undisturbed for several months. Large 50–100 µm diamond-shaped crystals formed (Supplementary Fig. S2) and were harvested after four months using various MicroLoop LD™ 50–200 μm (MiTeGen), and then flash frozen in liquid nitrogen without addition of cryoprotectant.

### Data collection and analysis

X-ray diffraction experiments were carried out on beamline 23-ID-B of the Advanced Photon Source at Argonne National Laboratory (Lemont, Illinois). Diffraction data were indexed using *xia2/DIALS*^54^ and scaled using AIMLESS^55^ from the CCP4i program package^56^. The acidic HeR structure was solved by molecular replacement with phaser^57^ using basic TaHeR^16^ as a search model (PDB ID 6is6). The model was manually built in Coot^58^ and iterative refinement was done with the phenix.refine routine of Phenix^59^. Refinement statistics are summarized in Table 1. Figures with 3D structures were prepared using PyMOL^53^.

### Data availability

The TaHeR structure at pH 4.5 was deposited to the Protein Data Bank under PDB ID 7u55.

### Phylogenetic tree

A multiple amino acid alignment was carried out using ClustalW^60^. The evolutionary distances were estimated by employing the Maximum Likelihood method and JTT matrix-based model^61^ using 100 bootstrap replicates. Evolutionary analyses were conducted in MEGA X^62^. The phylogenetic tree was rendered using iTOL^63^. The NCBI searchable sequence codes are provided in Supplementary Table S1.

### Site-directed spin-labelling of TaHeR-I51C

TaHeR-I51C protein expression was identical to the wildtype. However, the purification protocol was modified because the cysteine mutation introduced intermolecular disulfide bonds that formed covalent oligomers as shown by the two peaks in the size exclusion chromatogram (Supplementary Fig. S6). Thus, following Ni^2+^ IMAC purification, tenfold molar excess of the reducing agent 1,4-dithiothreitol (DTT) was added to TaHeR-I51C for 1 h at 4 °C in the dark, which successfully reduced disulfide bonds between Cys-51 residues. SEC was run on the reduced protein using a Superdex™ 200 10/300 GL column to remove the DTT and change the buffer to 20 mM MES, pH 7.2, 300 mM NaCl, 1% OG for optimal spin-labelling. Immediately after protein elution from the SEC, a tenfold molar excess of the spin label, either MTSL (1-oxyl-2,2,5,5-tetramethyl-∆3-pyrroline-3-methyl methanethiosulfonate) or IAP (3-(2-iodoacetamido) proxyl) was added and incubated for 2 h at 4 °C with gentle rotation to form TaHeR-I51R1 or TaHeR-I51P1. A second SEC was performed to remove excess label and unwanted residual covalent oligomers. The SEC fractions containing only the labelled protein dimer were then combined. See Supplementary Fig. S6 for a detailed overlay of the SEC spectra.

### Sample preparation for EPR

To prepare TaHeR-I51R1 or TaHeR-I51P1 for EPR, a PD MidiTrap G-25 column was employed to exchange the solvent to a basic buffer (pH 8, 20 mM Tris, 300 mM NaCl, 1% OG, D_2_O) or acidic buffer (pH 4.5, 20 mM sodium phosphate, 300 mM NaCl, 1% OG, D_2_O). D_2_O was used to increase the phase memory time and therefore the accessible time trace length for DEER experiments up to 7.0 μs^27–29^. Samples were concentrated to ~ 220 µM for TaHeR-I51P1, or ~ 110 µM for TaHeR-I51R1. 20% v/v D_8_-glycerol was added to the samples as a cryo-protectant for all subsequent CW-EPR and DEER experiments.

### EPR control experiments and CW-EPR

See supplementary methods, Fig. 7a, and Supplementary Fig. S7 for further details.

### DEER spectroscopy

High sensitivity Q-band (33.6–33.8 GHz) 4-pulse DEER experiments were conducted on a Bruker Elexsys E580 spectrometer equipped with a Super Q-FTu microwave bridge, a 10 W AmpQ and an EN 5107D2 Q-band flexline resonator. Spin-labelled samples (TaHeR-I51R1 or TaHeR-I51P1) were pipetted into quartz capillaries (1.5 mm inner diameter, 1.8 mm outer diameter, VitroCom) under dark or illuminated conditions. The dark samples were kept under light exclusion for 1 h before conducting measurements under dim red light (red LED lamps, λ_max_ ~ 625 nm). The light samples were illuminated with yellow light using a Fiber-Lite MI-150 lamp (Dolan-Jenner) equipped with a yellow filter (< 500 nm) for 60 s prior to flash freezing. The samples containing D_8_-glycerol as a cryoprotectant, were vitrified using a dry-ice/ethanol bath and stored at − 80 °C prior to data collection. The sample temperature was adjusted to 80 K with an Oxford Instruments continuous-flow cooling system and data collection was performed for 16–24 h to achieve optimum signal-to-noise ratios. A 32-ns π-pump pulse was applied to the low field peak of the nitroxide field swept spectrum, and the observer π/2 (16 ns) and π (32 ns) pulses were positioned 50–56 MHz (18–20 G) upfield. In order to cancel out receiver offsets and unwanted echoes, a 2-step phase cycle was employed. Distance distributions were obtained from the raw time traces using the Matlab®-based open-source program DeerAnalysis 2019^64^. To obtain the distance distributions P(r), a 3-D background correction was used exclusively for all data sets. After Tikhonov regularization, the GCV algorithm^65^ was chosen to determine an optimum regularization parameter (794 ≤ α ≤ 1000) from the resulting L curves. The Tikhonov validation error estimates of all P(r) are given in Supplementary Fig. S9. Theoretical DEER distance distributions of the TaHeR crystal structures and dimer-of-dimer models were calculated using the Matlab®-based, open-source program, Multiscale Modeling of Macromolecules (MMM)^66^. DeerAnalysis and MMM are both available at https://epr.ethz.ch/software.html. For all routines, Matlab® version R2020b was used.

### Dimer-of-dimer TaHeR models

Dimer-of-dimer HeR arrangements were acquired from all available HeRs crystal structures, Alphafold^51,52^, and using manipulation in PyMOL. Details are presented in the supplementary methods. All models are presented in Supplementary Fig. S10 and the best fit models are given in Fig. 7c.

### Allostery predictions

Allostery was analyzed using the rigidity-transmission allostery (RTA) algorithms^42,43,67^. The RTA method is based on mathematical rigidity theory^42,68^. Starting with TaHeR (PDB ID 7u55) and HeR 48C12 (PDB ID 6su4) dimers, a constrained network representation of protein structure was generated with the method FIRST^44^, which consists of nodes (atoms) and edges representing covalent and non-covalent interactions. The RTA was used to quantify the available conformational degrees of freedom at individual residues before and after perturbation of rigidity of the negatively charged ion (chloride or acetate) pocket occupying the SBC. The extent of the “degree of freedom transmission” was then extracted and visualized on the structure based on the strength of allosteric communication.

### Constrained geometric Monte Carlo simulations

To probe the dynamical features of TaHeR and HeR 48C12, we have applied a constrained geometric Monte Carlo simulation, based on methodology Framework Rigidity Optimized Dynamics Algorithm New (FRODAN)^68–70^. This approach, which can be regarded as a low computational complexity alternative to MD simulations, utilizes a coarse-grained molecular mechanics potential and rigidity theory to explore the wide regions of conformational space, probing the conformational ensemble well outside the starting structure. Starting with TaHeR and HeR 48C12 crystal structures, we first added hydrogen atoms using the MolProbity server, and ran FRODAN in the non-targeted mode, generating 30,000 candidate structures for each case. Simulations were carried out at different hydrogen bond energy cut-offs, from − 1.0 to − 2.0 kcal/mol; during each individual run, the cut-off value was kept constant. To evaluate the dynamics, backbone Root Mean Square Fluctuations (RMSF) were calculated.

## Supplementary Information

Supplementary Information.
