# Supplementary material for: Low pH structure of heliorhodopsin reveals chloride binding site and intramolecular signaling pathway
Source: Sci Rep. 2022 Aug 17;12:13955. doi: 10.1038/s41598-022-17716-9 (PMC9385722; doi:10.1038/s41598-022-17716-9)
Supplement: Supplementary file 1 — Supplementary Information. [file 41598_2022_17716_MOESM1_ESM.pdf]

Supporting Information

Low pH structure of heliorhodopsin reveals chloride binding site and intramolecular signaling pathway

Jessica E. Besaw [1,2], Jörg Reichenwallner [2], Paolo De Guzman [2,3], Andrejs Tucs [4], Anling Kuo [2], Takefumi Morizumi [2], Koji Tsuda [4,5,6], Adnan Sljoka [5,7], R. J. Dwayne Miller [1,8], and Oliver P. Ernst [2,9]

[1] Department of Chemistry, University of Toronto, Toronto, Ontario, M5S 3H6, Canada
[2] Department of Biochemistry, University of Toronto, Toronto, Ontario, M5S 1A8, Canada
[3] Department of Laboratory Medicine and Pathology, University of Toronto, Toronto, Ontario, M5S 1A8, Canada
[4] Graduate School of Frontier Sciences, The University of Tokyo, Kashiwa, Chiba 277-8561, Japan
[5] RIKEN Center for Advanced Intelligence Project, RIKEN, 1-4-1 Nihombashi, Chuo-ku, Tokyo 103-0027, Japan
[6] Research and Services Division of Materials Data and Integrated System, National Institute for Materials Science, Tsukuba, Ibaraki 305-0047, Japan
[7] Department of Chemistry, York University, Toronto, Ontario, M3J 1P3, Canada
[8] Department of Physics, University of Toronto, Toronto, Ontario, M5S 3H6, Canada
[9] Department of Molecular Genetics, University of Toronto, Toronto, Ontario, M5S 1A8, Canada

Supplementary Methods:

pH titration of the UV-Vis absorption maximum. Purified TaHeR from SEC (20 mM MES pH 6.5, 300 mM NaCl, and 1% OG) was concentrated to 3.5 mg/mL and 30-fold diluted with 0.1 M buffer (sodium acetate, MES, HEPES; selected for the desired pH), 100 or 300 mM NaCl, and 1% OG. The UV-Vis absorption spectra were measured in a quartz cuvette using a CARY 60 UV-Vis spectrophotometer equipped with a Peltier temperature control unit (Agilent Technologies) at 20 °C. Each sample was incubated in the sample holder for at least two minutes until the sample was dark-adapted before measurement between 250 nm – 750 nm. The λ_{max} values for each pH (Supplementary Fig. S3) were determined by averaging eight spectra for better signal-to-noise ratio, and analyzed by the IGOR Pro software (WaveMetrics, Lake Oswego, OR, USA).

Chloride binding at acidic pH. Purified TaHeR from SEC (20 mM Tris, pH 8, 300 mM NaCl, 1% OG) was chloride-depleted and acidified (20 mM sodium acetate, pH 5.0, 1% OG) via three runs through a PD MidiTrap G-25 desalting column, using 30 mL buffer washes between each run. 200 µL of 0.9 mg/mL TaHeR were prepared with known amounts of NaCl (0, 10, 50, 100 or 300 mM of NaCl) in buffer at pH 5.0 (20 mM sodium acetate, pH 5.0, 1% OG). The UV-Vis absorption spectra were measured in a quartz cuvette using a CARY 60 UV-Vis spectrophotometer at 20 °C. From the maximum λ at each chloride concentration $[\mathrm{Cl}^-]$, a binding curve was fitted to the data using Solver in Excel to determine an approximate dissociation constant ($\mathrm{K_d}$). In the following equation, λ_0 is the wavelength maximum at 0 mM of Cl^-, and λ_∞ is the wavelength maximum when all chloride binding sites are occupied.

$$\frac{(\lambda-\lambda_0)}{(\lambda_\infty-\lambda_0)} = \frac{[\mathrm{Cl}^-]}{[\mathrm{Cl}^-]+\mathrm{K_d}} \qquad \text{(S1)}$$

Continuous wave electron paramagnetic resonance (CW-EPR). X-band (~9.7 GHz) CW-EPR experiments were conducted on a Bruker Elexsys E500 spectrometer containing a Bruker ER 041 MR microwave bridge and an ER 4123D dielectric resonator. The samples (5 – 7 µL) were loaded into quartz capillaries (0.6 mm inner diameter, 0.84 mm outer diameter, VitroCom) via capillary action and sealed with Critoseal®. Data collection was performed at room temperature using a field sweep between 100 – 150 G, a modulation amplitude of 2 G, and a microwave power of 0.6346 mW. Samples were either measured in dark or light conditions. The dark samples were kept in the dark for 1 hour before conducting measurements under dim red light (red LED lamps, λ_{max} ~ 625 nm). The light samples were illuminated with yellow light using a Fiber-Lite MI-150 lamp (Dolan-Jenner) equipped with a 500 nm longpass filter (illuminating samples with >500 nm light) for 10 seconds prior to measurement, and throughout the entire duration of the data collection (Supplementary Fig. S7).

EPR control experiments. Two control experiments were performed to determine if Cys-168 and Cys-205 in wildtype TaHeR become spin-labelled upon addition of either MTSL or IAP. For the MTSL control experiment, purified TaHeR was labelled using the same procedure as TaHeR-I51C. CW-EPR revealed that after 30 minutes of room temperature incubation because no line broadening occurred in the TaHeR and MTSL mixed sample. The full removal of the unbound MTSL through SEC further only showed a very weak immobilized CW-EPR signal, even after concentrating the protein sample to 100 µM (Supplementary Fig. S7b). Therefore, the residual labeling efficiency of wildtype TaHeR can be considered as negligible. For the IAP control, purified wildtype TaHeR was labelled using the same procedure and concentration described for TaHeR-I51C. DEER experiments on IAP-incubated wildtype TaHeR showed no detectable dipolar modulations (Fig. 7a). Thus, the native cysteines in TaHeR are not sufficiently spin-labelled and do not contribute to our DEER-derived distance distributions from TaHeR I51C upon addition of MTSL or IAP. In this case, the DEER data set was analyzed using the program LongDistances by Christian Altenbach. The program is written in LabVIEW (National Instruments) and can be freely downloaded from the following site: http://www.biochemistry.ucla.edu/biochem/Faculty/Hubbell/.

Dimer-of-dimer TaHeR models. Dimer-of-dimer HeR arrangements were acquired from all available HeR crystal structures including HeR 48C12 and mutants (PDB IDs 6uh3, 6is6, 7clj, 6su3, 6su4, and 7u55). For each PDB structure, the extended crystal packing was generated from its space group symmetry in PyMOL to acquire tetramer

arrangements. Then, two TaHeR dimers (from the crystal structure at pH 4.5 or pH 8.0) were superimposed on these tetramer models, saved as a new structure, and used for further analysis by MMM[1].

Dimer-of-dimer assemblies were also generated from the artificial intelligence program, Alphafold[2,3], by inputting TaHeR amino acid sequence and selecting for tetramer arrangements. The program provided five dimer-of-dimer assemblies that were arranged in an L-shape or square shape. As before, the models were updated by superimposing two TaHeR X-ray dimers for further analysis in MMM.

To generate additional conformations, model manipulation was performed in PyMOL using the modified AlphaFold model 3 (‘square shaped’) as the starting model. In PyMOL, the tetramer was oriented in a top-down view, so the x-axis represents left/right, the y-axis is up/down, and the z-axis is coming out of the plane of the screen. To generate a staggered orientation, a 20 Å translation of a single dimer was applied along the y-axis. To generate a V-shaped orientation, each dimer was rotated by ±25° (50° total) along the z-axis, and then translated ±10 Å (20 Å total) along the x-axis to remove clashes. Dimer-of-dimer HeR assemblies are presented in Supplementary Fig. S10.

Intermolecular distance calculation. The following calculation shows that at 220 µM TaHeR, the average distance between TaHeR dimers at is 25 nm, or about four-fold greater than the largest peak distance at 6.6 nm.

$$\frac{\text{Dimers}}{\text{Volume}} = 220\ \mu\text{M TaHeR monomers} = 110\ \mu\text{M TaHeR dimers}$$
$$= \frac{110\ \times 10^{-6}\ \text{mol dimers}}{\text{L}}\left(\frac{6.022\ \times 10^{23}\ \text{molecules}}{\text{mol}}\right)\left(\frac{1\ \text{L}}{10^{24}\ \text{nm}^3}\right) = \frac{6.62\ \times 10^{-5}\ \text{dimers}}{\text{nm}^3}$$

$$\frac{\text{Volume}}{\text{dimer}} = \left(\frac{6.62\ \times 10^{-5}\ \text{dimers}}{\text{nm}^3}\right)^{-1} = \frac{15096\ \text{nm}^3}{\text{dimer}}$$

$$\frac{\text{Distance}}{\text{Dimer}} = \frac{(\text{Volume})^{\frac{1}{3}}}{\text{dimer}} = \frac{(15096\ \text{nm}^3)^{\frac{1}{3}}}{\text{dimer}} = 25\ \text{nm}$$

Table S.1. Protein sequences used in the phylogenetic tree. Table continued on the next page.

Abbreviation	Protein Full Name	Organism	NCBI searchable code	Type 1 or HeR	Function
VirR$_{DTS}$ (PgV VirR)	DTS-motif rhodopsin from *Phaeocystis globosa* virus 12T	Virus	6JO0_A (YP_008052647.1)	Type 1	H+ pump
OLPVRII	Organic Lake Phycodnavirus rhodopsin II	Virus	6SQG_A	Type 1	Na+/K+ selective ion channel, can also be an outward H+ pump
GPR	Green-light absorbing proteorhodopsin	Bacteria	Q9F7P4.1	Type 1	Outward H+ pump
BPR	Blue-light absorbing proteorhodopsin	Bacteria	Q9AFF7.2	Type 1	Outward H+ pump
XR	*Salinibacter ruber* Xanthorhodopsin	Bacteria	CBH24619.1	Type 1	Outward H+ pump
GR	*Gloeobacter* rhodopsin (Gloeobacter violaceus PCC 7421)	Bacteria	BAC88139.1	Type 1	Outward H+ pump
TR	Thermophilic rhodopsin (*Thermus thermophilus* JL-18 bacteriorhodopsin)	Bacteria	AFH39233.1	Type 1	Outward H+ pump
FR	*Fulvimarina pelagi* rhodopsin	Bacteria	WP_007065598.1	Type 1	Inward Cl- pump
NmClR (NM-R3)	*Nonlabens marinus* S1-08 Cl-pumping Rhodopsin	Bacteria	5G54_A	Type 1	Inward Cl- pump
PoClR	*Parvularcula oceani* Cl- pumping Rhodopsin	Bacteria	WP_051881608.1	Type 1	Inward Cl- pump (ClR)
KR2	*Krokinobacter eikastus* rhodopsin 2	Bacteria	4XTO_A	Type 1	Outward Na+ pump
GlNaR (GLR)	*Gillisia limnaea* rhodopsin (sodium pumping rhodopsin)	Bacteria	WP_040506994.1	Type 1	Outward Na+ pump
PoNaR	*Parvularcula oceani* Na+ pumping Rhodopsin	Bacteria	WP_051881578.1	Type 1	Outward Na+ pump
CfRh-PDE1	*Choanoeca flexa* rhodopsin phosphodiesterase 1	Eukaryota	QDH43407.1	Type 1	Enzymerhodopsin (light dependent PDE activity)
CfRh-PDE4	*Choanoeca flexa* rhodopsin phosphodiesterase 4	Eukaryota	QDH43410.1	Type 1	Enzymerhodopsin (light dependent PDE activity)
SrRh-PDE	*Salpingoeca rosetta* rhodopsin phosphodiesterase	Eukaryota	EGD79054.1	Type 1	Enzymerhodopsin (light dependent PDE activity)
HvSRII	*Haloarcula vallismortis* Sensory rhodopsin II	Archaea	BAM76970.1	Type 1	Sensor
HsSRII	*Halobacterium salinarum* Sensory rhodopsin II	Archaea	AAC44370.1	Type 1	Sensor
HvSRI	*Haloarcula vallismortis* Sensory rhodopsin I	Archaea	EMA00853.1	Type 1	Sensor
HsSRI	*Halobacterium salinarum* Sensory rhodopsin I	Archaea	QCC45373.1	Type 1	Sensor
PoXeR	*Parvularcula oceani* xenorhodopsin	Bacteria	WP_051881467.1	Type 1	Inward H+ pump
NsXeR	Candidatus Nanosalina sp. J07AB43 xenorhodopsin	Archaea	EGQ43296.1	Type 1	Inward H+ pump
RmXeR	*Rubricoccus marinus* xenorhodopsin	Bacteria	WP_094549673	Type 1	Inward H+ pump
HsHR	*Halobacterium salinarum* halorhodopsin	Archaea	QCC44078.1	Type 1	Inward Cl- pump
NpHR	*Natronomonas pharaonis* halorhodopsin	Archaea	AAA72222.1	Type 1	Inward Cl- pump
SrHR	*Salinibacter ruber* M8 halorhodopsin	Bacteria	CBH25915.1	Type 1	Inward Cl- pump
HsBR, BR	*Halobacterium salinarum* bacteriorhodopsin	Archaea	1C3W_A	Type 1	Outward H+ pump
AR1	*Halorubrum sodomense* archaerhodopsin-1	Archaea	P69052.1	Type 1	Outward H+ pump
AR2	*Halorubrum sodomense* archaerhodopsin-2	Archaea	AAB19870.2	Type 1	Outward H+ pump

Abbreviation	Protein Full Name	Organism	NCBI searchable code	Type 1 or HeR	Function
AR3	*Halorubrum sodomense* archaerhodopsin-3	Archaea	BAA09452.1	Type 1	Outward H+ pump
ChR1	*Chlamydomonas reinhardtii* channel rhodopsin 1	Eukaryota	ACD70142.1	Type 1	Cation Pump (H^+, Na^+, K^+, Ca^{2+})
ChR2	*Chlamydomonas reinhardtii* channel rhodopsin 2	Eukaryota	ABO64386.1	Type 1	Cation Pump
ACR1	*Guillardia theta* anion channelrhodopsin-1	Eukaryota	AKN63094.1	Type 1	Anion pump
ACR2	*Guillardia theta* anion channelrhodopsin-2	Eukaryota	AKN63095.1	Type 1	Anion pump
LaSzR-2	*Lokiarchaeota* archaeon schizorhodopsin 2	Archaea	QBQ84358.1	Type 1	Inward H^+ pump
AntR	Antarctic Rhodopsin	Bacteria	Ga0105045_102227662	Type 1	Inward H^+ pump
SzR4	Schizorhodopsin-4	Archaea	7E4G_A	Type 1	Inward H^+ pump
VPS401HeR	*Emiliania huxleyi* virus PS401 heliorhodopsin	Virus	AET73409.1	HeR	proton transport activity
V2HeR3	*Emiliania huxleyi* virus 202 heliorhodopsin 3	Virus	AET42421.1	HeR	light gated proton channel
McHeR	*Micromonas commoda* heliorhodopsin	Eukaryota	XP_002509300.1	HeR	no ion transport activity
V2HeR2	*Emiliania huxleyi* virus 202 heliorhodopsin 2	Virus	AET42570.1	HeR	no ion transport
V2HeR1	*Emiliania huxleyi* virus 202 heliorhodopsin 1	Virus	AET42597.1	HeR	no ion transport
V1HeR1	*Emiliania huxleyi* virus 201 heliorhodopsin 1	Virus	AET97940.1	HeR	no ion transport
V1HeR2	*Emiliania huxleyi* virus 201 heliorhodopsin 2	Virus	AET97964.1	HeR	no ion transport
HULAa30F3	Uncultured Dehalococcoidia bacterium clone HULAa30F3 Heliorhodopsin	Bacteria	MW122882.1 (QOV09072.1)	HeR	no ion transport, may be sensor with neighbour DegV
HULAa36F11	Uncultured Thermoplasmata archaeon clone HULAa36F11	Archaea	MW122884.1 (QOV09127.2)	HeR	no ion transport
HULAa50H9	Candidatus *Nanopelagicales* bacterium clone HULAa50H9 Heliorhodopsin	Bacteria	MW122877.1 (QOV08958.1)	HeR	no ion transport
HULAa45C8S	Uncultured *Micrococcales* bacterium clone HULAa45C8S Heliorhodopsin	Bacteria	MW122878.1 (QOV08962.1)	HeR	no ion transport
HULAa55C9	Uncultured actinobacterium clone HULAa55C9 heliorhodopsin	Bacteria	MW122876.1 (QOV08887.1)	HeR	no ion transport
EINA20F1	Candidatus *Nanopelagicales* bacterium clone EINA20F1 Heliorhodopsin	Bacteria	MW122873.1 (QOV08797.1)	HeR	no ion transport
HULAa3G5	Uncultured *Micrococcales* bacterium clone HULAa3G5	Bacteria	MW122881.1 (QOV09024.1)	HeR	no ion transport
HULAa2F4	Uncultured *Micrococcales* bacterium clone HULAa2F4 Heliorhodopsin	Bacteria	MW122880.1 (QOV08993.1)	HeR	no ion transport
HULAb132A11	Candidatus *Nanopelagicales* bacterium clone HULAb132A11	Bacteria	MW122875.1 (QOV08884.1)	HeR	no ion transport
TaHeR	*Thermoplasmatales archaeon* SG8-52-1 heliorhodopsin	Archaea	KYK26602.1	HeR	no ion transport
BcHeR	*Bellilinea caldifistulae* heliorhodopsin	Bacteria	WP_061912808	HeR	no ion transport
HeR 48C12	Heliorhodopsin from marine fosmid 48C12	Bacteria	AVZ43932.1	HeR	no ion transport
HbHeR	*Halolactibacillus* (Multispecies) heliorhodopsin	Bacteria	WP_062321037.1	HeR	spectroscopy only
DaHeR	*Dehalogenimonas alkenigignens* heliorhodopsin	Bacteria	WP_058438211.1	HeR	spectroscopy only

Abbreviation	Protein Full Name	Organism	NCBI searchable code	Type 1 or HeR	Function
MiHeR	*Mesotoga inferea* heliorhodopsin	Bacteria	WP_169700581.1	HeR	spectroscopy only
TcHeR	*Thermococcus* sp. 2319×1 Heliorhodopsin	Archaea	WP_175060008.1	HeR	spectroscopy only
HtHeR	*Halorhabdus tiamatea* SARL4B heliorhodopsin	Archaea	WP_198408551.1	HeR	spectroscopy only
NdHeR	*Nocardioides dokdonensis* heliorhodopsin	Bacteria	WP_068108470.1	HeR	spectroscopy only
SmHeR	*Streptomyces* sp. CC77 heliorhodopsin	Bacteria	WP_071268943.1	HeR	spectroscopy only
SbHeR	*Salinibacterium* sp. PAMC 21357 heliorhodopsin	Bacteria	WP_010205440.1	HeR	spectroscopy only
SxHeR	*Salinibacterium xinjiangense* heliorhodopsin	Bacteria	WP_097060109.1	HeR	spectroscopy only
DlHeR	*Demequina lutea* heliorhodopsin	Bacteria	WP_062073994.1	HeR	spectroscopy only
KaHeR	*Knoellia aerolata* heliorhodopsin	Bacteria	WP_035936430.1	HeR	spectroscopy only
OpHeR	*Ornithinimicrobium pekingense* heliorhodopsin	Bacteria	WP_022919890.1	HeR	spectroscopy only
AbHeR	Actinobacteria bacterium IMCC26103 heliorhodopsin	Bacteria	QLL25365.1	HeR	spectroscopy only
MfHeR	*Methanobacterium formicicum* heliorhodopsin	Archaea	WP_048085542	HeR	uncharacterized
PfHeR	*Pyrococcus furiosus* heliorhodopsin	Archaea	WP_011011843.1	HeR	uncharacterized
HpHeR	*Halolamina pelagica* heliorhodopsin	Archaea	WP_189319165.1	HeR	uncharacterized
HrHeR	*Halosimplex rubrum* heliorhodopsin	Archaea	WP_179909546.1	HeR	uncharacterized
HuHeR	*Halorhabdus utahensis* heliorhodopsin	Archaea	WP_015789609.1	HeR	uncharacterized

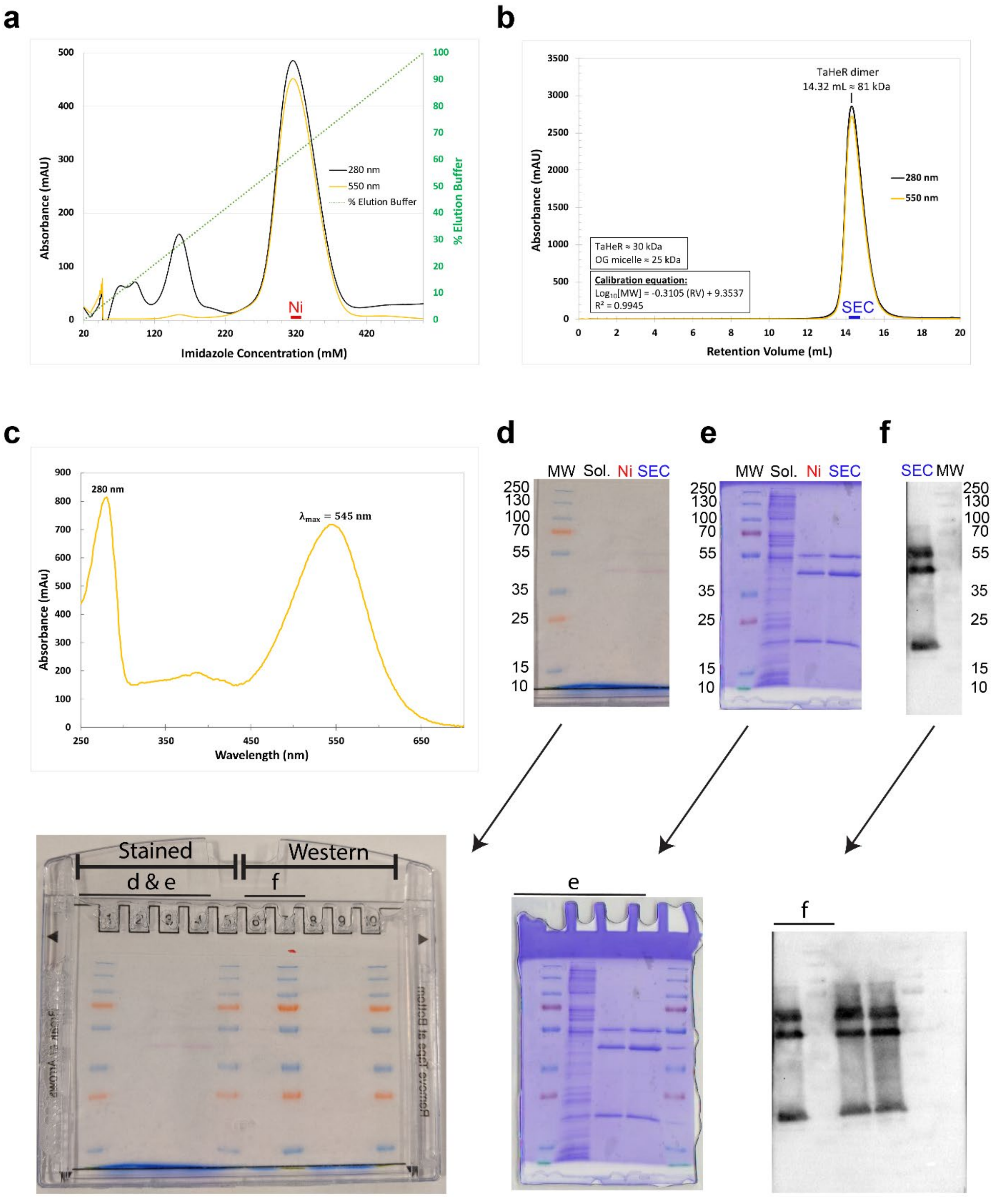

Figure S1. Expression, purification, and characterization of *Thermoplasmatales* archaeon SG8-52-1 heliorhodopsin (TaHeR). (a) IMAC elution profile. TaHeR is eluted from a Ni^{2+} NTA affinity column with a linear imidazole gradient (20 to 500 mM). TaHeR elutes between 275 – 375 mM imidazole. (b) Size exclusion chromatography suggests TaHeR is a dimer at pH 6.5 in OG detergent. TaHeR is eluted from a Superdex^{TM} 200 10/300 GL column using buffer (10 mM MES, pH 6.5, 300 mM NaCl, 1% OG). The elution was monitored at 280 nm and 550 nm to reveal protein and

heliorhodopsin, respectively. A single coinciding peak at 14.32 mL suggests pure, homogenous TaHeR protein. Using SEC protein calibration standards (Cytiva), the elution peak was determined to be 81 kDa. This would suggest TaHeR oligomerizes as a dimer (2 x 30 kDa; 30 kDa molecular weight of TaHeR) within an OG detergent micelle (25 kDa[4]) (c) UV-vis absorption spectrum of purified TaHeR in buffer (10 mM MES, pH 6.5, 300 mM NaCl, 1% OG). The maximum absorbance at pH 6.5 occurs at λ_{max} = 545 nm. SDS-PAGE of (d) unstained gel and (e) Coomassie-stained gel, and (f) an anti-His western blot, which was run on the same gel. Three bands are observed, at approximately 20, 40 and 55 kDa, all corresponding to TaHeR as identified by western blotting. The pink color of the 40 and 55 kDa bands demonstrated the presence of retinal in stable TaHeR oligomers, even in the presence of SDS detergent. Arrows point to the original, uncropped version of the gels.

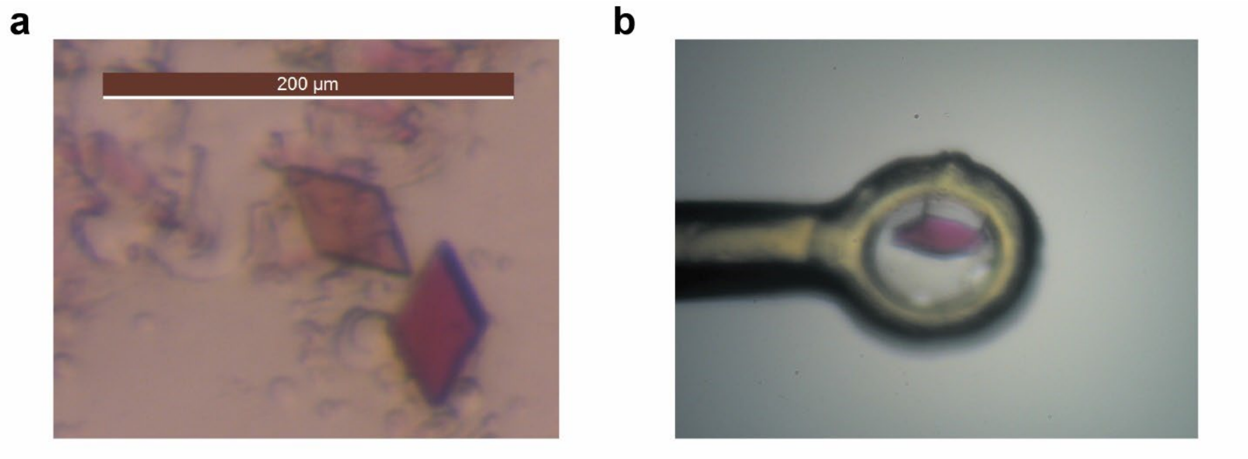

Figure S2. (a) Bicelle crystallization of TaHeR yields diamond-shaped crystals. (b) TaHeR crystal in a 50 µm Mitegen MicroLoop prior to X-ray diffraction collection on beamline 23-ID-B of the Advanced Photon Source at Argonne National Laboratory (Lemont, Illinois).

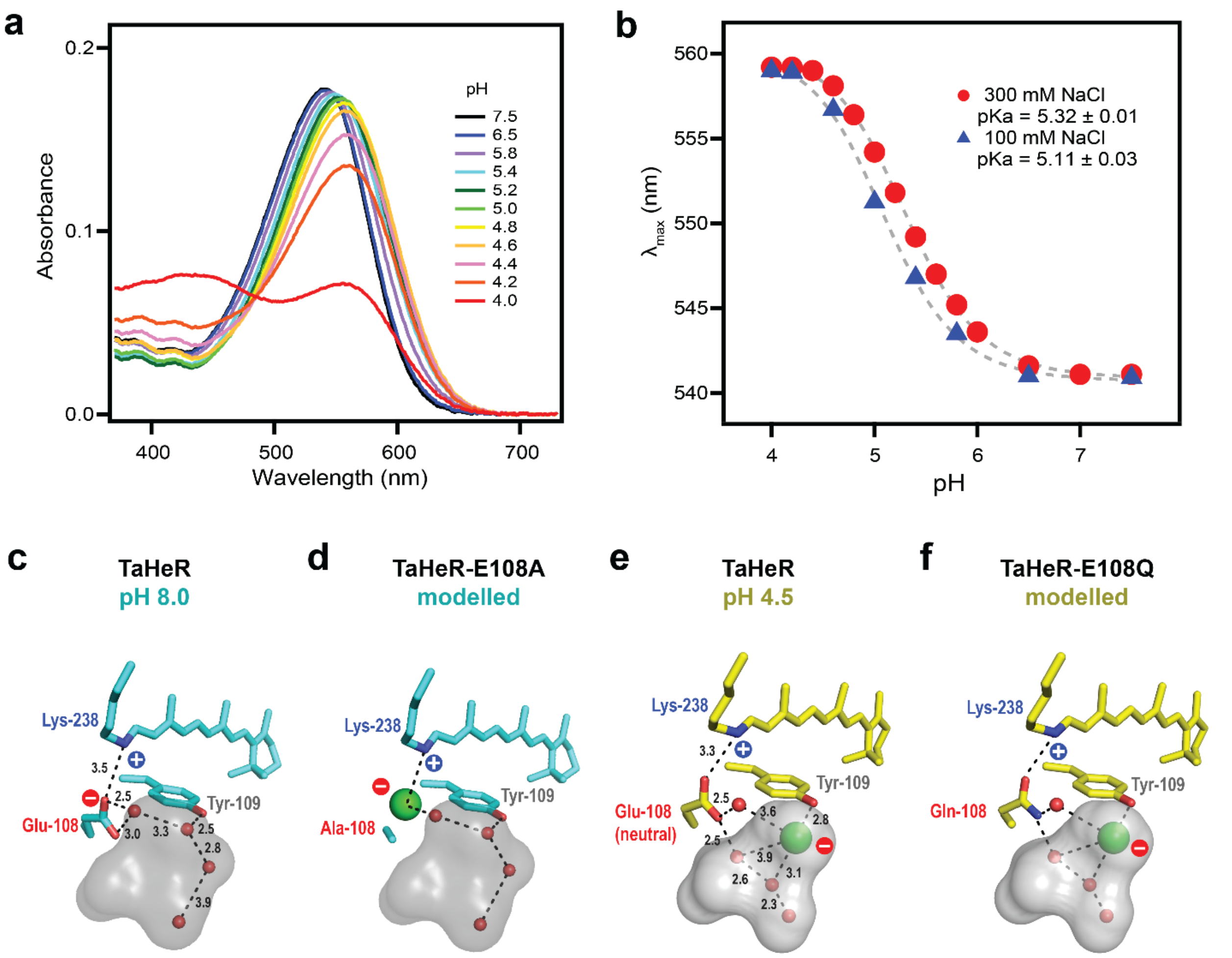

Figure S3. pH-dependent red shift of the UV-Vis absorption maximum of TaHeR in octyl glucoside (OG) and corresponding TaHeR structural models. (a) Visible absorption spectra of wildtype TaHeR at pH 4.0 – 7.5 measured in a 0.1 M buffer (acetate, MES, or HEPES; selected for pH), 300 mM NaCl, 1% OG. (b) pH titration of TaHeR shows an 18 nm acid-induced red shift in the presence of 100 mM NaCl (blue triangles) and 300 mM NaCl (red circles), presumably due to protonation of the retinal Schiff base counterion, Glu-108. The pK_a values indicate mean ± standard deviation for eight replicate measurements. The pK_a of Glu-108 is 5.11 in 100 mM NaCl and 5.32 in 300 mM NaCl. Previously, Shihoya *et al.* reported TaHeR Glu-108 to have a pK_a of 3.6 in dodecyl maltoside (DDM) detergent and 100 mM NaCl[5]. Although counterion protonation is known to correlate with the chloride binding of TaHeR, lowering NaCl to 100 mM showed little effect on pK_a in OG detergent, suggesting that the pK_a was greatly shifted probably due to the increased flexibility of the protein in OG. We assume a similar pK_a shift towards higher pH under our crystallization condition. (c-f) TaHeR structural models of the $RSBH^+$ shown in (c) wildtype at pH 8 (PDB ID 6is6), (d) E108A, modelled based on the pH 8 structure (e) wildtype at pH 4.5 (PDB ID 7u55), and (f) TaHeR-E108Q, modelled based on the pH 4.5 structure. Distances are reported in Å. The SBC is shown as a grey surface. Panel (d) and (f) were originally proposed for HeR 48C12 in Singh *et al.*[6]

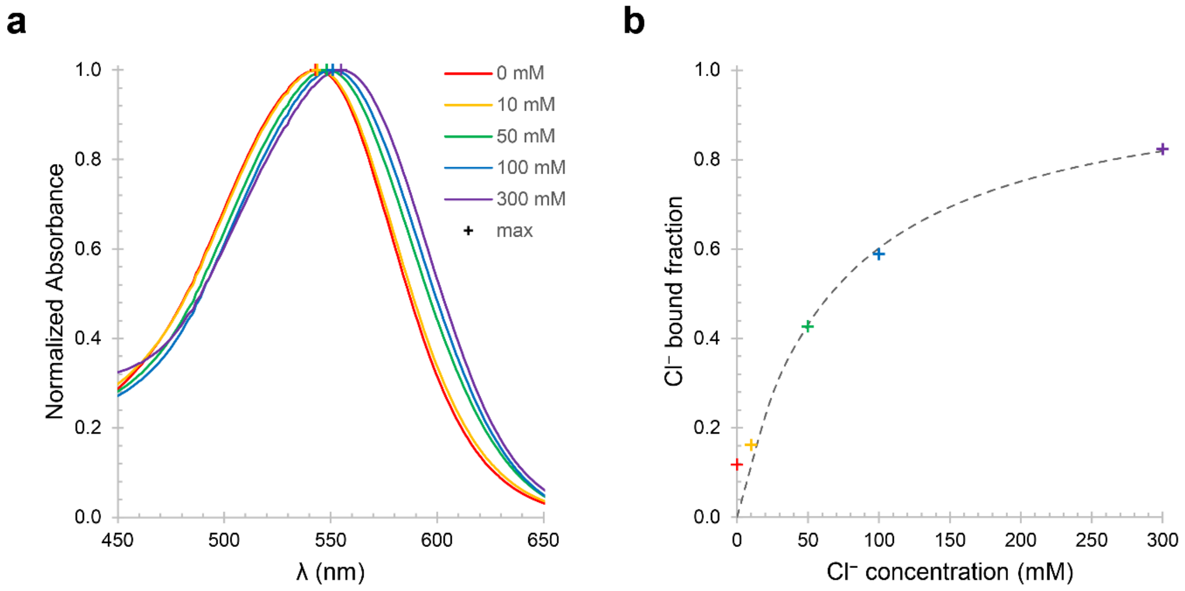

Figure S4. Chloride binding to TaHeR at pH 5. (a) Normalized UV-vis absorbance spectra of purified TaHeR upon addition of NaCl (0, 10, 50, 100, or 300 mM) reveals chloride-induced red shift in the λ_{max} from 543 nm to 555 nm under acidic conditions. The acidic buffer was 20 mM sodium acetate, pH 5, 1% OG. The λ_{max} value in each curve is indicated with a cross. (b) Estimated chloride-bound fraction at a given chloride concentration. The dashed line shows the fit curve according to equation S1 (see above). In this equation, we approximate 0% chloride-bound at $\lambda_0 \approx 541$ nm and 100% chloride-bound at $\lambda_\infty \approx 558$ nm (from Supplementary Fig. S3), with a resulting equilibrium constant of K_d = 67 mM.

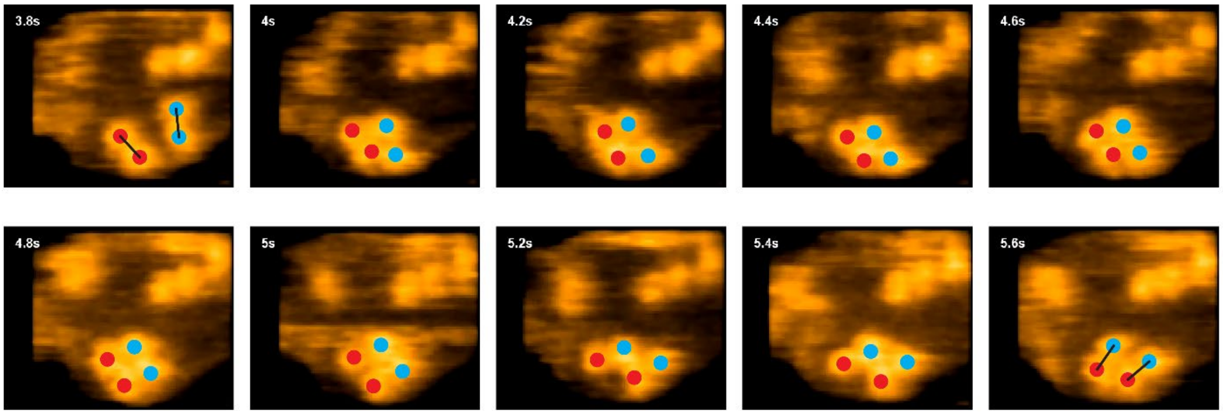

Figure S5. HS-AFM images of HeR 48C12 from the supplementary videos of ref.[5] showing protomer exchange by forming dimer-of-dimer assemblies. Red and blue circles have been added as a guide to show the exchange from only red or blue dimers (connected by a black line) to red-blue mixed dimers. Reprinted by permission from CCC: Springer Nature, Shihoya *et al.* (2019)[5].

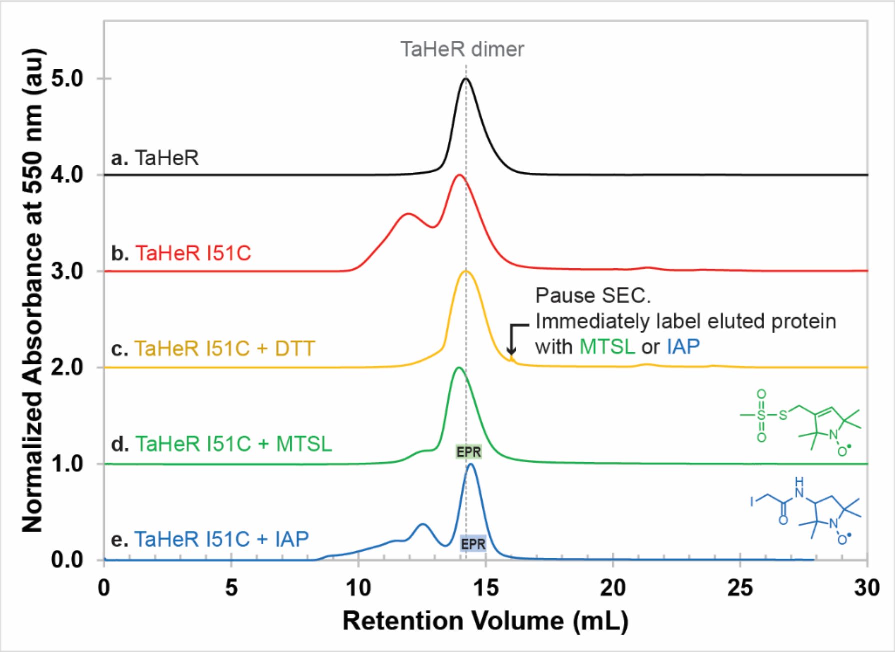

Figure S6. Purification and spin-labelling of TaHeR I51C as monitored via size exclusion chromatography (SEC). Retinal-bound protein absorbance at 550 nm was monitored as a function of retention volume. (a) A comparative TaHeR wildtype profile shows a single peak eluting at ~14.2 mL representing the TaHeR dimer. (b) TaHeR I51C forms two elution bands encompassing the dimer and a larger oligomer (~12.0 mL). The larger oligomer is likely formed via intermolecular disulfide bonds between the Cys-51 side chains. (c) Incubation of TaHeR-I51C with the reducing agent DTT (at 10X protein concentration) for 2 hours successfully reduces the unwanted disulfide bonds and removes the larger oligomer. Note that the running buffer does not contain DTT, and the purpose of this SEC is to remove the DTT for subsequent spin-labelling. An arrow marks the point where, directly after elution, the protein fractions are combined with spin label. (d) Incubation of TaHeR-I51C with MTSL for 2 hours followed by SEC to remove excess spin label yields predominantly dimers with a small shoulder at 12.5 mL. Green bar shows the fractions combined for generating EPR samples. Structure of MTSL shown as green sticks. (e) Incubation of TaHeR-I51C with the spin label IAP for 2 hours followed by SEC to remove excess spin label forms a major dimer peak and two minor elution peaks at 12.5 mL and 11.5 mL. Blue bar shows the fractions combined for EPR samples. Structure of IAP shown as blue sticks. All purifications were run on a Superdex^{TM} 200 10/300 GL column using running buffer 20 mM Tris, pH 7.2, 300 mM NaCl, 1% OG. 0.8 mL – 1 mL TaHeR was loaded for each sample.

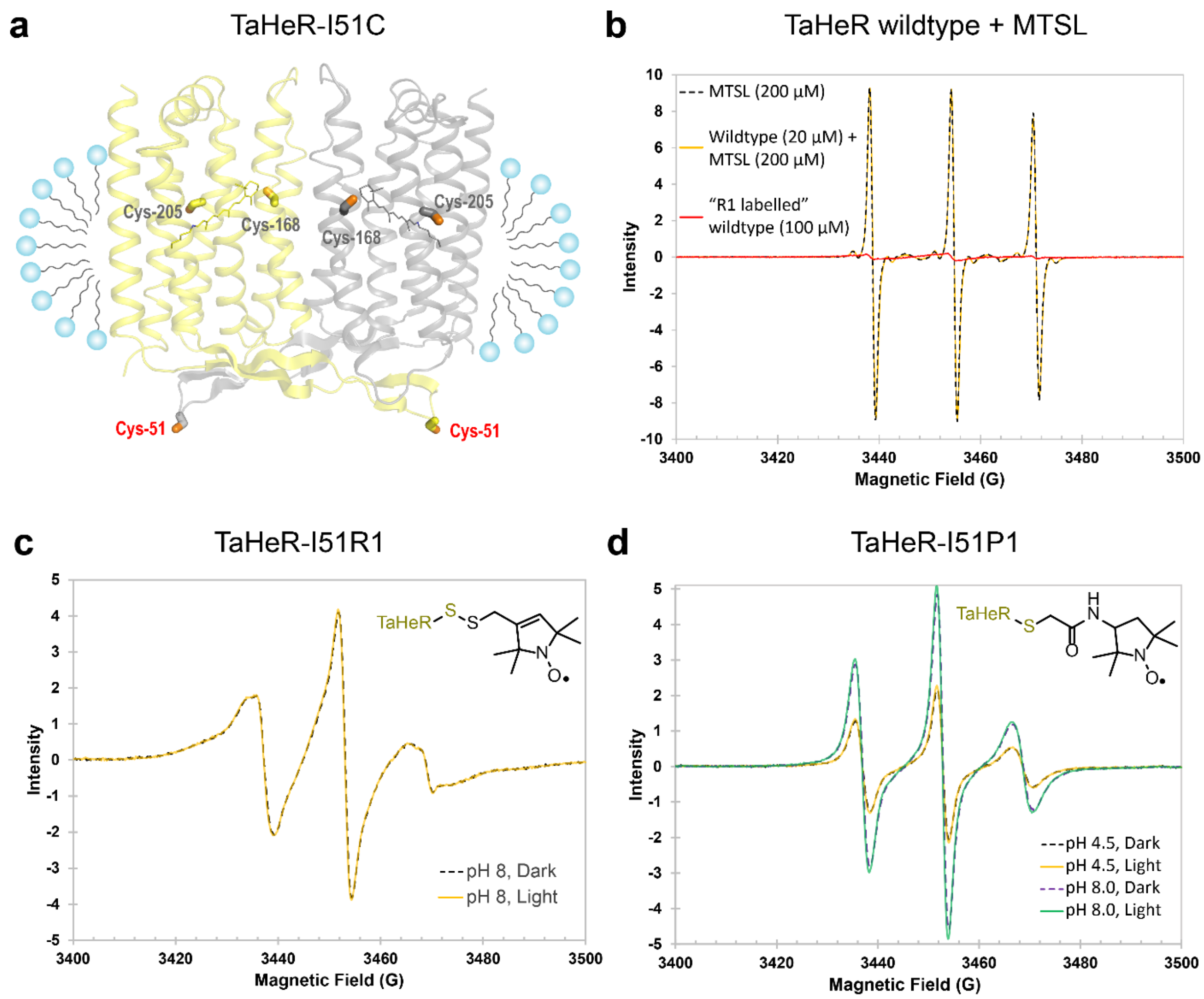

Figure S7. CW-EPR spectra of spin-labelled TaHeR and TaHeR-I51C. CW-EPR reveals wildtype TaHeR is not spin-labelled upon addition of MTSL, while mutant TaHeR-I51C is successfully spin-labelled with MTSL (forming TaHeR-51R1) or IAP (forming TaHeR-51P1). (a) Model of TaHeR-I51C dimer in detergent micelle (blue sphere with stem). Wildtype TaHeR has two cysteines in transmembrane domain, Cys-205 and Cys-168 (grey label), and the TaHeR-I51C mutation introduces Cys-51 (red label) at the apex of the A–B loop. Cysteines are shown as sticks with the sulfur highlighted in orange and retinal chromophore shown as thin lines. (b) CW-EPR spectra from 20 μM of purified wildtype TaHeR with 200 μM of MTSL (yellow) compared to 200 μM MTSL label only (black dash) shows lack of peak broadening. Primarily, this reveals that no cysteines are labelled. Size exclusion column of the MTSL + wildtype TaHeR sample to remove unbound MTSL results in the "R1 labelled" wildtype spectrum (red, 100 uM), which further demonstrates negligible labelling of the native cysteines (less than ca. 5% labelling efficiency). All samples were present in a 20 mM Tris, pH 7.2, 300 mM NaCl, 1% OG buffer. TaHeR and MTSL were mixed at room temperature for 30 minutes prior to acquiring spectra. CW-EPR spectra of purified (c) TaHeR-I51R1 and (d) TaHeR-I51P1 (see main text for purification details). The dark samples were kept in the dark for 1 hour before conducting measurement under dim red light. The light samples were illuminated with yellow light for 10 seconds prior to measurement, and throughout the entire data collection. The protein concentration was 110 μM for TaHeR-I51R1 and 220 μM for TaHeR-I51P1. The basic buffer at pH 8 was 20 mM Tris, 300 mM NaCl, 1% OG. The acidic buffer at pH 4.5 was 20 mM sodium phosphate, 300 mM NaCl, 1% OG.

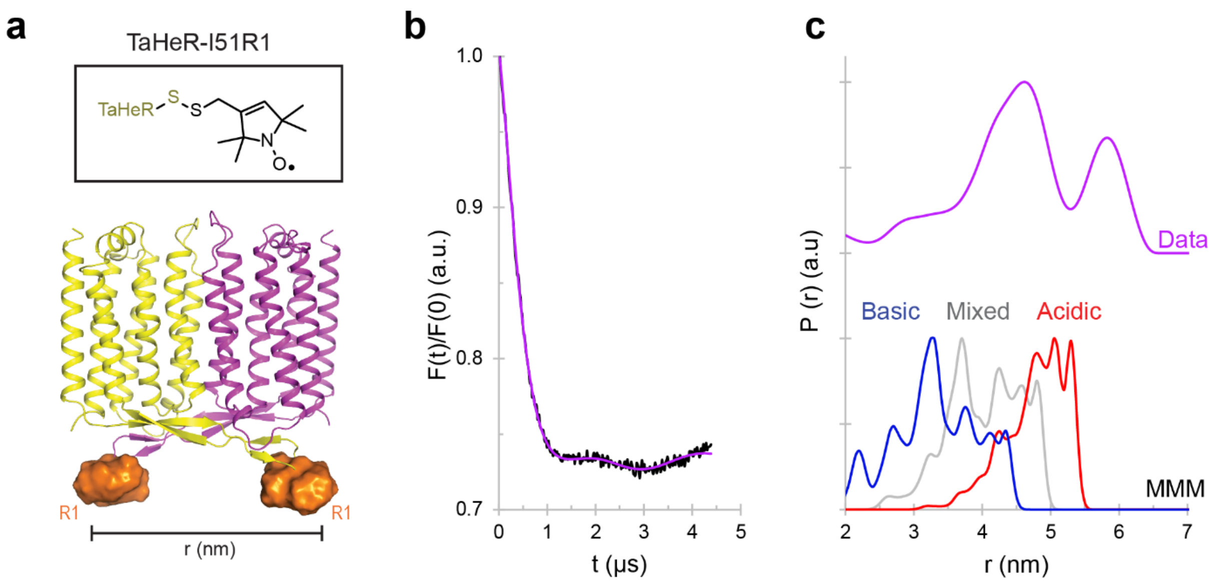

Figure S8. Exemplary DEER experiment of TaHeR-I51R1. (a) Model of the TaHeR-I51R1 dimer with R1 rotamer distribution (orange surface) from the pH 4.5 crystal structure. The distance r between R1 spin labels is indicated schematically. Inset shows the structure of a covalently bound R1 label (box). (b) The background corrected dipolar evolution function of TaHeR-I51R1 at pH 8. Time traces up to t_{max} = 4.5 µs yield reliable distance distributions of up to 5.2 nm (and mean distances up to 6.5 nm).[#] (c) DEER-derived distance distribution P(r) for 110 µM TaHeR-I51R1 at pH 8.0 (purple curve) shows two main peaks at 4.6 nm and 5.8 nm. Computed MMM distance distribution plots based on the pH 4.5 (blue), pH 8.0 (red), and mixed (grey, i.e. a protomer of pH 8.0 and pH 4.5 in the dimer) crystal structure shows a shift towards larger distances with decreasing pH but does not sufficiently explain the long 5.8 nm peak. The buffer at pH 8.0 was 20 mM Tris, 300 mM NaCl, 1% OG, D_2O, 20% D_8-glycerol.

[#] The reliability of the distance distribution scales with the maximum dipolar evolution function time, t_{max}.[7] Quantitatively, the mean distances (r_m in nm) and distribution widths are credible up to $r_m = 4\ \mathrm{nm} \cdot \sqrt[3]{\frac{t_{max}}{2}}$, while only mean distances (and not distribution widths) are reliable up to $r_m = 5\ \mathrm{nm} \cdot \sqrt[3]{\frac{t_{max}}{2}}$.[7,8]

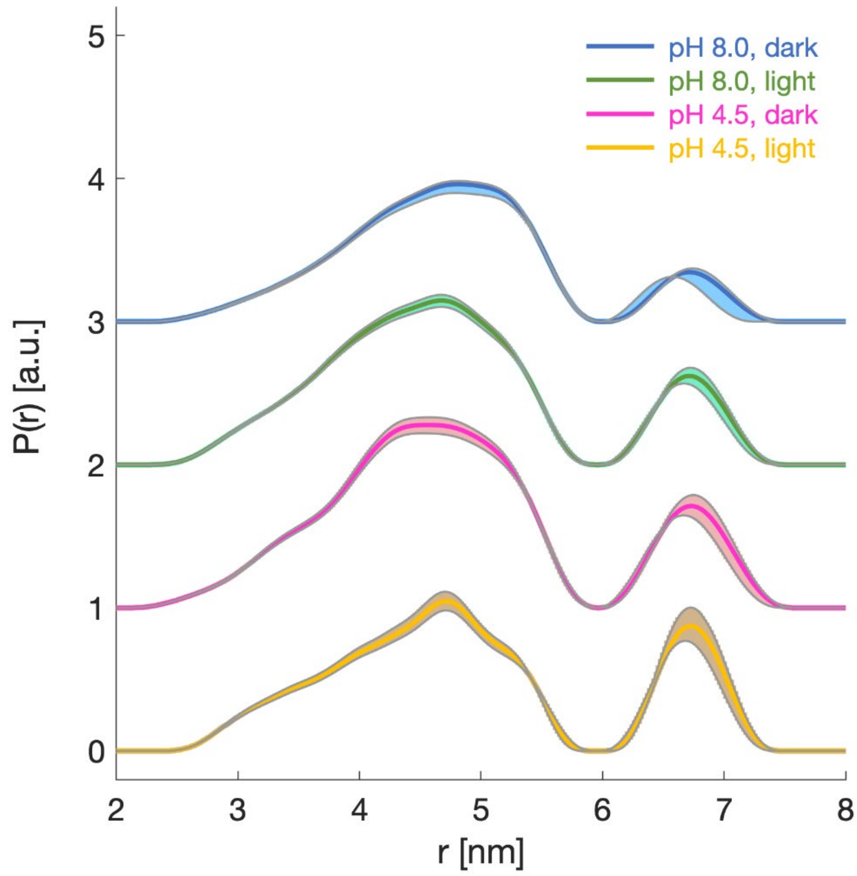

Figure S9. DEER distance distribution P(r) of TaHeR-I51P1 including Tikhonov validation. The shaded curves represent the error intervals of the distribution curves. For validation in DeerAnalysis2019, the background correction was screened in the range from 1600 ± 800 ns and modulation depth (Δ) was varied by ± 5%. Background dimensionalities of D = 3.0 gave most reliable and stable results throughout all four data sets.

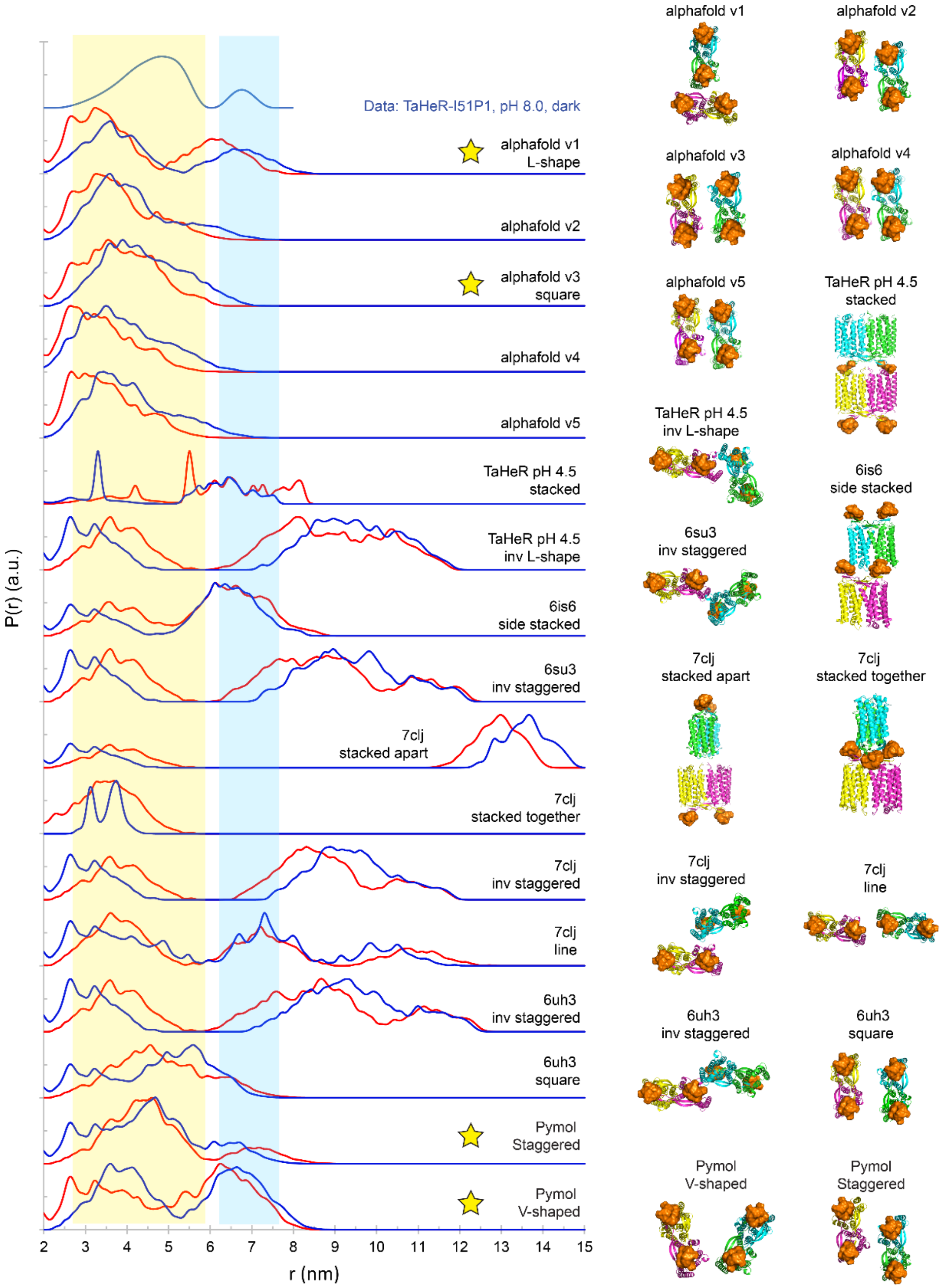

Figure S10. MMM distance distribution for TaHeR-I51P1 dimer-of-dimers assemblies generated from AlphaFold, HeR X-ray structures (PDB ID: 6is6, 6su3, 6uh3, 7clj, 6u55) or PyMOL, compared to the DEER distance distribution for TaHeR-I51P1 at pH 8.0 in the dark (top blue curve). The protomers in all generated tetramers have been replaced with TaHeR at pH 4.5 (red), or 8.0 (dark blue, PDB ID 6is6). Duplicate conformations have been removed (i.e. PDB ID 6su3 ≈ 6su4). The stars highlight conformations that are included in the main manuscript (Fig. 7c).

References

1 Polyhach, Y., Bordignon, E. & Jeschke, G. Rotamer libraries of spin labelled cysteines for protein studies. *Phys Chem Chem Phys* **13**, 2356-2366, doi:10.1039/c0cp01865a (2011).

2 Jumper, J. *et al.* Highly accurate protein structure prediction with AlphaFold. *Nature* **596**, 583-589, doi:10.1038/s41586-021-03819-2 (2021).

3 Varadi, M. *et al.* AlphaFold Protein Structure Database: massively expanding the structural coverage of protein-sequence space with high-accuracy models. *Nucleic Acids Res* **50**, D439-D444, doi:10.1093/nar/gkab1061 (2022).

4 Stetsenko, A. & Guskov, A. An Overview of the Top Ten Detergents Used for Membrane Protein Crystallization. *Crystals* **7**, 197 (2017).

5 Shihoya, W. *et al.* Crystal structure of heliorhodopsin. *Nature* **574**, 132-136, doi:10.1038/s41586-019-1604-6 (2019).

6 Singh, M., Katayama, K., Béjà, O. & Kandori, H. Anion binding to mutants of the Schiff base counterion in heliorhodopsin 48C12. *Phys Chem Chem Phys* **21**, 23663-23671, doi:10.1039/c9cp04102h (2019).

7 Jeschke, G. DEER distance measurements on proteins. *Annu Rev Phys Chem* **63**, 419-446, doi:10.1146/annurev-physchem-032511-143716 (2012).

8 Schmidt, T., Walti, M. A., Baber, J. L., Hustedt, E. J. & Clore, G. M. Long Distance Measurements up to 160 A in the GroEL Tetradecamer Using Q-Band DEER EPR Spectroscopy. *Angew Chem Int Ed Engl* **55**, 15905-15909, doi:10.1002/anie.201609617 (2016).
